# New Data Indicate Larger Decline in Morphological Diversity in Split-Footed Lacewing Larvae than Previously Estimated

**DOI:** 10.3390/insects16020125

**Published:** 2025-01-27

**Authors:** Laura Buchner, Simon Linhart, Florian Braig, Gideon T. Haug, Thomas Weiterschan, Carolin Haug, Joachim T. Haug

**Affiliations:** 1 Faculty of Biology, Biocenter, Ludwig-Maximilians-Universität München (LMU Munich), Großhaderner Str. 2, 82152 Planegg-Martinsried, Germany; laura.buchner@campus.lmu.de (L.B.); simon.linhart@palaeo-evo-devo.info (S.L.); florian.braig@palaeo-evo-devo.info (F.B.); gideon.haug@palaeo-evo-devo.info (G.T.H.); carolin.haug@palaeo-evo-devo.info (C.H.); 2 Independent Researcher, Forsteler Str. 1, 64739 Höchst im Odenwald, Germany; thomas.weiterschan@web.de; 3 GeoBio-Center at LMU, Richard-Wagner-Str. 10, 80333 München, Germany

**Keywords:** biodiversity, Myanmar amber, Neuroptera, Nymphidae, quantitative morphology

## Abstract

Modern insect decline sparks interest in similar declines of the past. Neuroptera, the group of lacewings, has been suggested to have undergone decline after the Cretaceous, and quantitative studies of larval morphology have supported this view. One of the groups within Neuroptera seeing a major decline is that of split-footed lacewings, Nymphidae. We here report 13 new larval specimens of split-footed lacewings from about 100-million-year-old Kachin amber, Myanmar. We explore how enlarging the data set to 60 specimens and expanding the investigated morphological structures affect the recognition of decline. The expanded data set has an increased morphospace, indicating a lack of saturation. The decline is, therefore, likely even larger than so far anticipated for Nymphidae. The study of head and mouthparts provides the strongest signal for a loss of diversity, which is feasible due to the direct coupling to feeding ecology. Analysis of the trunk region cannot recover a signal for loss of diversity. Sampling issues play a role here: the trunk of the larvae is less often well preserved, and the fossil larvae are more likely in early developmental stages due to the constraints of preservation.

## Simple Summary

Nymphidae, also known as split-footed lacewings, represent an ancient group within lacewings that has experienced a notable decrease in diversity over time. Through the examination of fossil specimens preserved in 100-million-year-old Myanmar amber, our aim was to collect quantitative data on this decline. An analysis, encompassing newly reported larvae in addition to previously documented ones, disclosed that the morphological diversity of larvae of Nymphidae was considerably greater in the past compared to the present. By comparing the morphology of these ancient larvae with their contemporary counterparts, extinct morphologies were identified, indicating a loss of ecological diversity across geological epochs. This diminishment in diversity echoes patterns observed in other lacewing groups, suggesting a broader trend of ecological transformation among these insects. The findings illuminate the historical ecology of lacewing larvae and underscore the significance of understanding past biodiversity dynamics to inform conservation endeavors in the current era.

## 1. Introduction

According to different studies, the diversity of the group Insecta appears to be declining in recent times, usually either measured via species numbers or biomass [1,2,3,4,5,6,7,8,9,10,11]. This decline influences many important ecological functions (including ecosystem services) as these are fulfilled by numerous representatives of Insecta [12,13,14], among them beetles, bees, and flies. Concerning ecological functions, the larval phase of these animals can be considered the most important life phase, as the larva is the phase of growing and feeding [15]. In many groups within Insecta, more lifetime is spent as a larva than as an adult, with sometimes even non-feeding and extremely short-lived adults [16,17]. This pattern is especially present in Holometabola, which is a group within Insecta containing the so-called “big four” [18]: Coleoptera (beetles) [19,20,21], Diptera (e.g., flies) [22,23,24], Lepidoptera (e.g., butterflies) [25,26,27], and Hymenoptera (e.g., wasps) [28,29,30]. Each of these four groups has more than 100,000 formally described species. Holometabolans have strongly specialized larval stages that differ significantly from their adult counterparts, and their larvae are most likely part of every terrestrial [31] and freshwater ecosystem [32,33].

Neuroptera, the group of lacewings and also part of Holometabola, is, however, less species-rich, with only about 6000 formally described species in the modern fauna [34,35,36,37,38,39,40,41]. Yet, the group has been assumed to have been more diverse in the past [35,42].

Therefore, lacewings seem to be ideal for investigating losses and declines in diversity over time. To better understand the role of larvae and how they are related to the processes of decline, studying lacewing larvae seems especially worthwhile. Fortunately, there is a good record of fossil lacewing larvae, mostly preserved in ambers of different ages (e.g., [43,44,45,46,47,48,49,50,51,52,53,54,55,56,57,58,59,60,61,62,63,64,65,66,67,68,69,70,71,72,73,74,75]).

Different lacewing groups have already been studied to determine a possible loss of larval diversity. For this purpose, quantitative morphological aspects have been used as a proxy, which can reveal differences undetectable with taxonomic comparisons [71]. According to these studies (see [71] and references therein), some lineages have lost diversity, others have not changed in diversity or provided inconclusive results, and few provided indications of later diversification. Yet, overall, the group has clearly declined in terms of the morphological diversity of the larvae over the last 100 million years [71]. One of the lineages with clear losses in larval diversity is that of Nymphidae, the group of split-footed lacewings [69,70]. There are only 35 described extant species, restricted to Australia and New Guinea [76].

Nymphidae is a group within Myrmeleontiformia or antlion-like lacewings; the latter also includes the famous antlions, of which some of the larvae dig a funnel into the soil to trap their prey, especially ants [77,78]. Antlion-like larvae ancestrally bear teeth on the stylets (specialized mouthparts formed by the upper and lower jaw, i.e., mandible and maxilla) [79,80]. The number of teeth varies among the groups within antlion-like lacewings [43].

Larvae of split-footed lacewings, in contrast, can usually be well recognized by the stylets bearing a single prominent tooth [43,81]. In the modern fauna, larvae of split-footed lacewings can be further separated into two forms: larvae of Nymphinae and Myiodactylinae. Larvae of Nymphinae are more elongated, often carry a “trash” packet (for camouflaging), and usually live on the ground [79,81,82,83]. Larvae of Myiodactylinae, in contrast, have a more disc-shaped trunk (at least in later stages), do not carry a “trash” packet, and usually live on trees [35,79,83,84,85,86,87,88,89,90].

Fossil larvae related to modern split-footed lacewings have been reported from Eocene Baltic amber [43] and especially Cretaceous Kachin amber from Myanmar [64,68,69,70,75]. In this study, 13 new fossil specimens are additionally reported, which are interpreted as related to modern split-footed lacewings. We performed several outline analyses of different morphological structures to investigate the morphological diversity among the previously known and the new larvae of Nymphidae. The results showed that the new specimens expanded the morphological diversity of the Cretaceous fauna and influenced the perception of the decline in Nymphidae since that time.

## 2. Materials and Methods

### 2.1. Material

Directly studied specimens (13 in total) are all preserved in approximately 100 million-year-old Kachin amber from Myanmar. Numerous specimens were purchased via the trading platform ebay from the traders burmitefossil and burmite-miner. Specimens are deposited in the Palaeo-Evo-Devo Research Group Collection of Arthropods, Ludwig-Maximilians-Universität München (LMU Munich), Germany, under repository numbers PED 0856, 1294, 1437, 1777, 2211, 2242, 2626, 2627, and 2770. Further specimens originate from the collection of one of the authors (TW; BuB 4, 12, 19, 29). Additional specimens included in further analyses come from literature (details below; Supplementary Table S1). The performed analyses included different numbers of specimens, depending on the different data sets: stylet and head: 44; stylet: 48; head: 48; body with stylet: 25; body without stylet: 29; head, prothorax, and stylet: 25; head and prothorax: 29; prothorax: 28; trunk: 29; trunk without prothorax: 29. In total, of 51 different specimens, at least 1 outline could be used for the analyses.

### 2.2. Imaging and Documentation

Directly examined samples for this study were documented on a VHX 6000 digital microscope (Keyence, Osaka, Japan). Amber pieces were mounted in a Petri dish with modeling clay at desired angles. Each amber piece was covered with a drop of glycerol and a cover slip to obtain an even and clear surface. The specimens were photographed with high-dynamic-range imaging (HDR) using several lenses, providing magnification from 20× up to 2000×, under unpolarised ring light and cross-polarised coaxial light, on a white and black background, and additionally transmitted light. Each image is a composite image from several single images (frames) with various focus layers, which were fused and then stitched together as a panorama image to obtain an in-depth, high-resolution image. The specimens were photographed from the dorsal and ventral sides if both were accessible. Images providing the best contrast were processed further.

### 2.3. Image Processing and Presentation

Adobe Photoshop CS 2 and CS3 were used to optimize color saturation and sharpness. Visible structures were color-marked and labeled to provide an interpretation of the accessible structures.

### 2.4. Measurements

Specimens were measured using the open-source software Fiji 2.0.0 (or ImageJ 1.53) [91]. The total body length of the larvae (including the stylets) or the accessible body length was measured.

### 2.5. Outlines

Outlines of up to ten different structures were considered to explore the full range of morphological diversity, all in dorsal or ventral view: stylet, entire body (head and trunk) with stylet, entire body without stylet, body without head (trunk), body without head and prothorax, head with stylet, head without stylet, prothorax with head and stylet, prothorax with head, and prothorax. The body outlines exclude locomotory appendages (legs); body parts were artificially straightened. Stylets were proximally amended with a half-circle. All outlines were drawn in Adobe Illustrator or Inkscape. Outlines were either based on images in this publication or from the literature [35,43,60,64,69,70,75,79,81,82,83,84,85,87,89,92].

### 2.6. Shape Analysis

Shape analysis was performed using the program SHAPE, which performs an elliptic Fourier transformation and a principal component analysis, in general following the method of Iwata and Ukai [93]. Two analyses experienced difficulties in alignment in SHAPE and, therefore, were conducted using the Momocs package (ver. 1.3.2) [94] in the R-statistics environment (ver. 4.1.0; R Core Team 2021).

The two most important dimensions (principal components), PC1 and PC2, which represent the components with the largest and second-largest variation in the data set, were plotted against each other. Values were plotted using OpenOffice Calculator, then re-drawn and visualized using different versions of Adobe Photoshop.

## 3. Results

### 3.1. Description of New Fossil Larvae

The numbering of the documented new specimens in this study is a continuation of the data set assembled in earlier studies [69,70,75].

**Figure 1:**
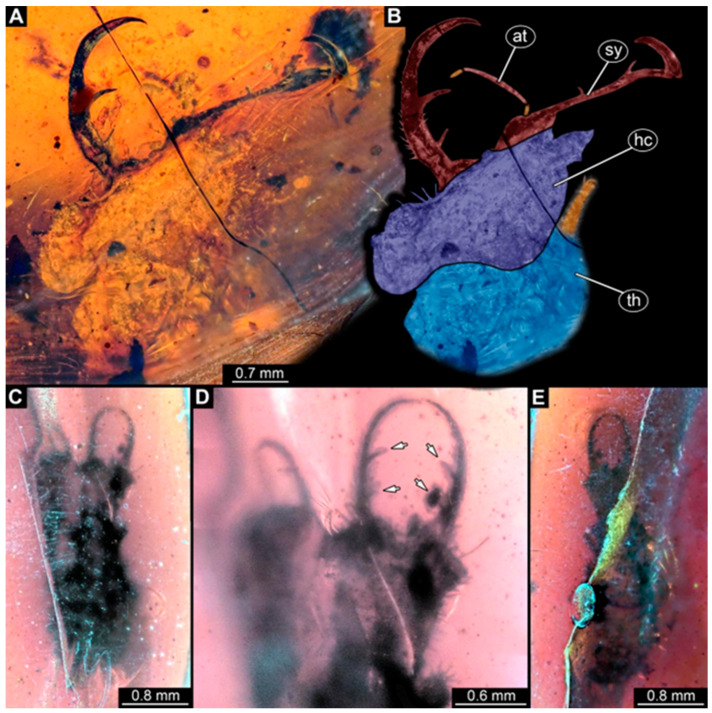
Specimens in Myanmar amber. (**A**,**B**) Specimen 246 (PED 1294). (**A**) Ventral or dorsal view. (**B**) Ventral or dorsal view, color-marked. (**C**–**E**) Specimen 244 (PED 0856). (**C**) Ventral or dorsal view. (**D**) Head capsule in ventral or dorsal view; arrows mark teeth. (**E**) Ventral or dorsal view. Abbreviations: at = antenna; hc = head capsule; sy = stylet; th = thorax.

**Figure 2:**
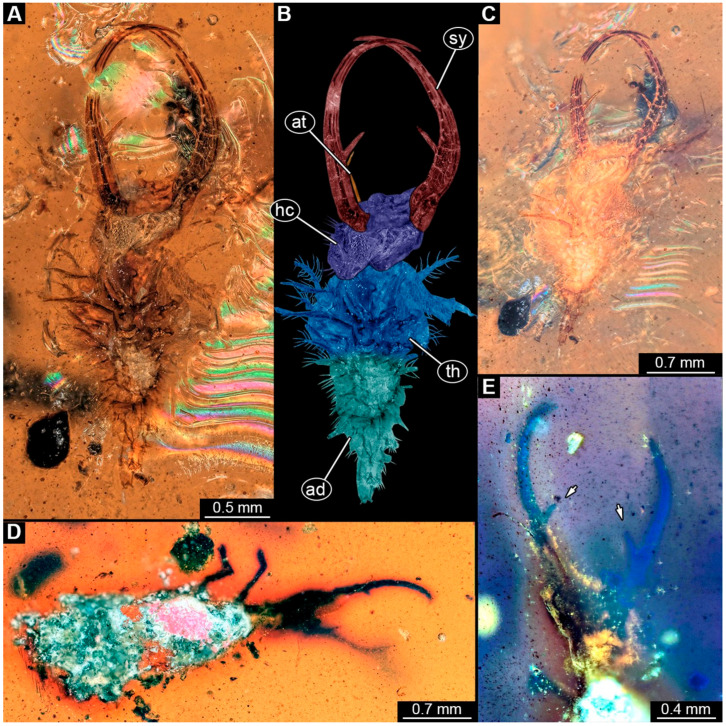
Specimens in Myanmar amber. (**A**–**C**) Specimen 250 (PED 2211). (**A**) Ventral or dorsal view. (**B**) Ventral or dorsal view, color-marked. (**C**) Ventral or dorsal view; cross-polarized light. (**D**,**E**) Specimen 247 (PED 1437). (**D**) Ventral or dorsal view. (**E**) Head capsule in ventral or dorsal view; arrows mark teeth. Abbreviations: ad = abdomen; at = antenna; hc = head capsule; sy = stylet; th = thorax.

**Figure 3:**
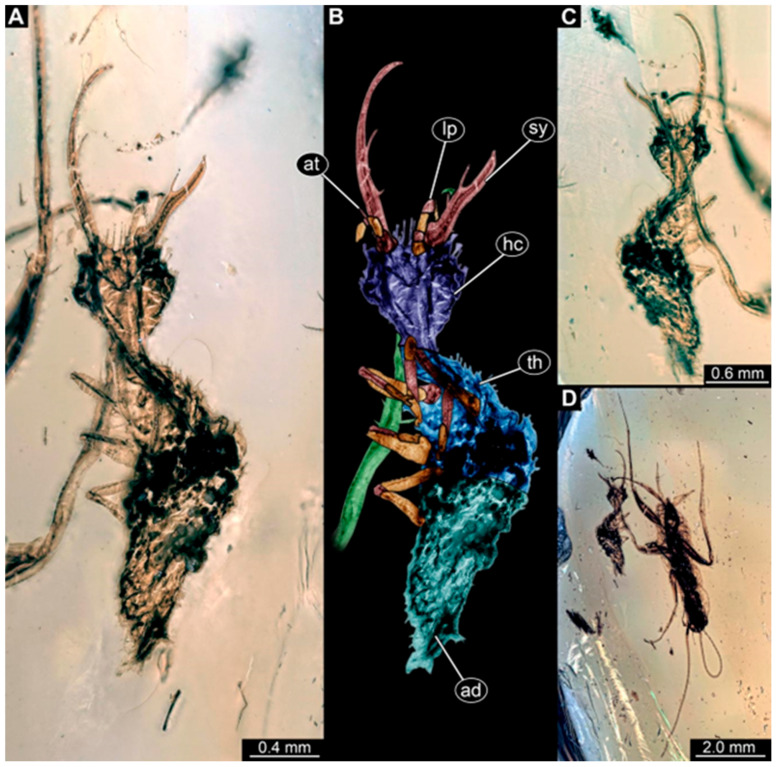
Specimen 249 (PED 1777); Myanmar amber. (**A**) Ventral view. (**B**) Ventral view, color-marked. (**C**) Dorsal view. (**D**) Syninclusion with different insects. Abbreviations: ad = abdomen; at = antenna; hc = head capsule; lp = labial palp; sy = stylet; th = thorax.

**Figure 4:**
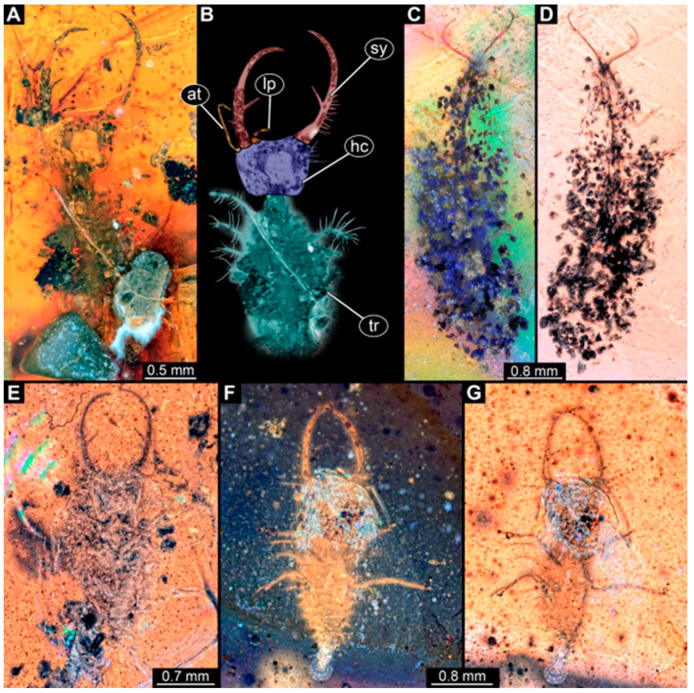
Specimens in Myanmar amber. (**A**,**B**) Specimen 251 (PED 2242). (**A**) Ventral or dorsal view. (**B**) Ventral or dorsal view, color-marked. (**C**,**D**) Specimen 257 (BuB 29). (**C**) Ventral or dorsal view. (**D**) Ventral or dorsal view. (**E**) Specimen 253 (PED 2626) in ventral or dorsal view. (**F**,**G**) Specimen 254 (PED 2627). (**F**) Ventral or dorsal view; black background. (**G**) Ventral or dorsal view; white background. Abbreviations: at = antenna; hc = head capsule; lp = lapial palp; sy = stylet; tr = trunk.

**Figure 5:**
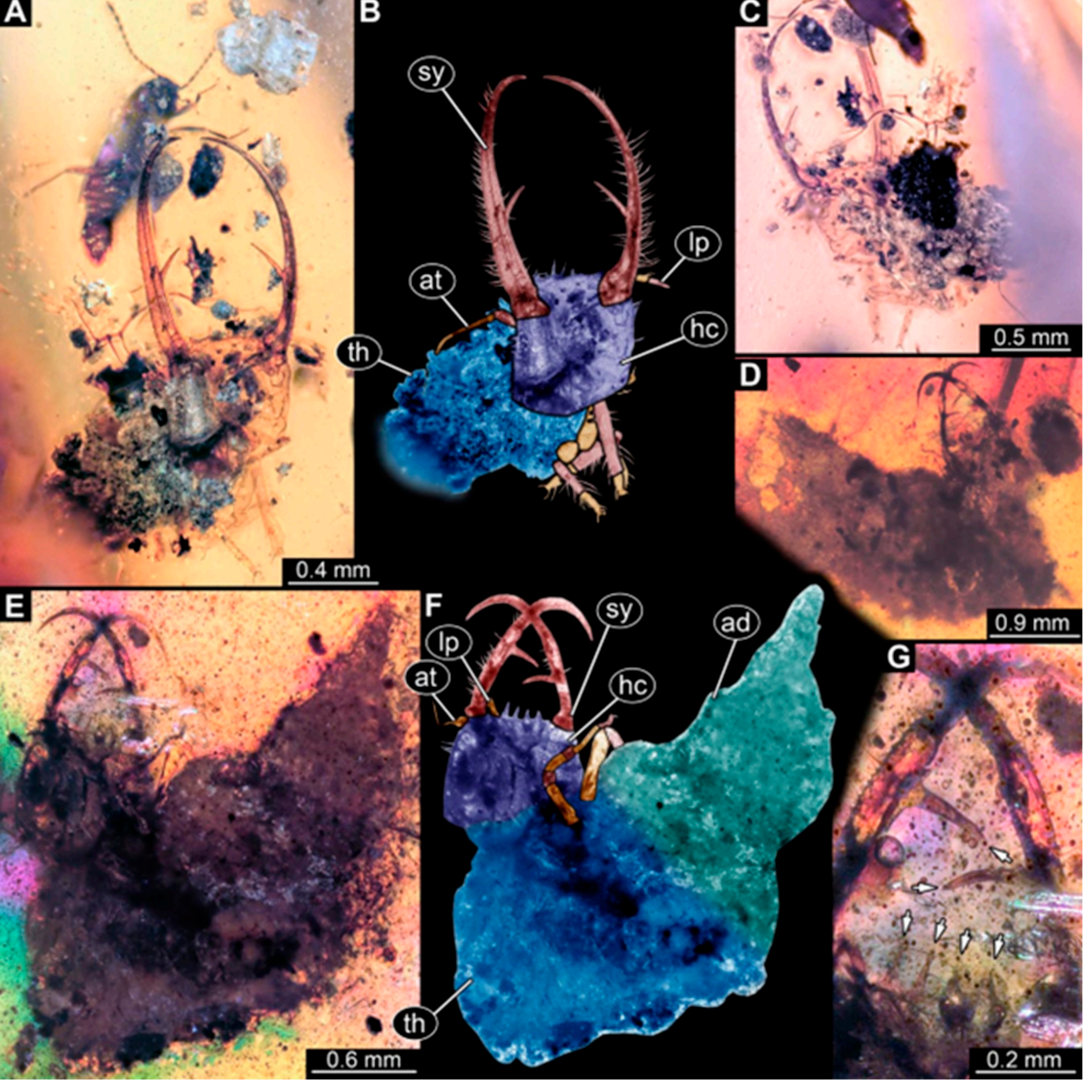
Specimens in Myanmar amber. (**A**–**C**) Specimen 255 (BuB 4). (**A**) Dorsal view. (**B**) Dorsal view, color-marked. (**C**) Ventral view. (**D**–**G**) Specimen 256 (BuB 19). (**D**) Dorsal view. (**E**) Ventral view. (**F**) Ventral view; color-marked. (**G**) Close-up of stylet and head capsule; arrows mark teeth and protrusions. Abbreviations: ad = abdomen; at = antenna; hc = head capsule; lp = labial palp; sy = stylet; th = thorax.

**Figure 6:**
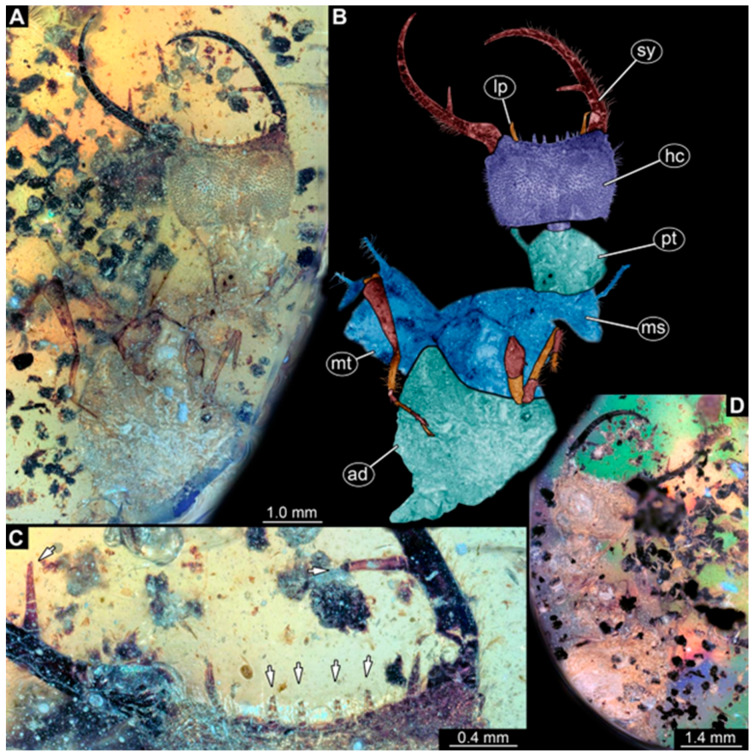
Specimen 258 (BuB 12); Myanmar amber. (**A**) Ventral or dorsal view. (**B**) Ventral or dorsal view; color-marked. (**C**) Close-up of stylet and head capsule; arrows mark teeth and protrusions. (**D**) Ventral or dorsal view. Abbreviations: ad = abdomen; hc = head capsule; lp = labial palp; ms = mesothorax; mt = metathorax; pt = prothorax; sy = stylet.

**Figure 7:**
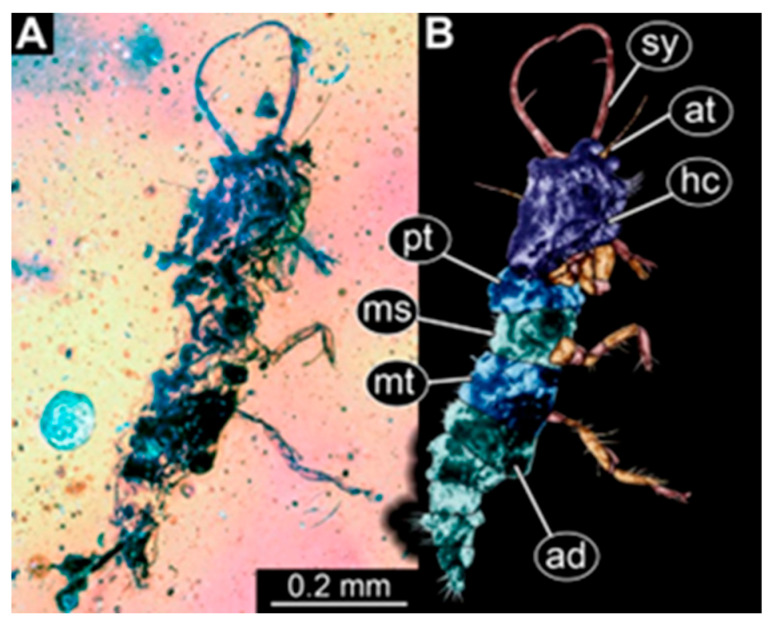
Specimen 260 (PED 2770); Myanmar amber. (**A**) Ventral or dorsal view. (**B**) Ventral or dorsal view, color-marked. Abbreviations: ad = abdomen; at = antenna; hc = head capsule; ms = mesothorax; mt = metathorax; pt = prothorax; sy = stylet.

(1) Specimen 244 (PED 0856) is largely concealed in the ventral and dorsal view by cloudiness, dirt particles, and flaws in the amber (Figure 1C,E). Each stylet seems to bear one longer and one shorter tooth (Figure 1D).
(2) Specimen 246 (PED 1294) is largely concealed by flaws in the amber in the ventral or dorsal view (Figure 1A,B). Each stylet bears one longer forward-inward-curved tooth and a shorter distally pointing tooth. The trunk seems to be almost completely missing. The specimen has a length of about 3.9 mm.
(3) Specimen 247 (PED 1437) is largely concealed by cloudiness and dirt particles in the ventral or dorsal view; only the rough outline is apparent (Figure 2D). Each stylet seems to bear a single tooth (Figure 2E). The specimen has a length of about 4.5 mm.
(4) Specimen 249 (PED 1777) is well accessible in the ventral (Figure 3A,B) and dorsal view (Figure 3C,D). Each stylet bears one longer forward-inward-curved tooth and a shorter distally pointing tooth. Four protrusions are visible on the anterior rim of the head. A syninclusion with a different insect (probably Orthoptera) is present (Figure 3D). The specimen has a length of about 3.5 mm.
(5) Specimen 250 (PED 2211) is well accessible in the ventral or dorsal view (Figure 2A–C). Each stylet bears one slightly forward-inward-curved tooth. Protrusions (possible scoli) are visible at some trunk segments. The specimen has a length of about 4.5 mm.
(6) Specimen 251 (PED 2242) is largely concealed in the ventral or dorsal view by cloudiness and dirt particles (Figure 4A,B). Each stylet bears one rather straight tooth. A rather slender cervix seems to be present. The posterior trunk seems to be missing. The specimen has a length of about 3.6 mm.
(7) Specimen 253 (PED 2626) is largely concealed by cloudiness and dirt particles in the ventral or dorsal view; only the rough outline is apparent (Figure 4E). Each stylet seems to bear a single tooth. The specimen has a length of about 3.0 mm.
(8) Specimen 254 (PED 2627) is largely concealed by cloudiness and dirt particles in the ventral and dorsal view; only the rough outline is apparent (Figure 4F,G). Each stylet seems to bear a single tooth. The specimen has a length of about 3.9 mm.
(9) Specimen 255 (BuB 4) is accessible in the dorsal (Figure 5A,B) and ventral view (Figure 5C) but partly concealed by dirt and cloudiness in the dorsal and ventral view. Each stylet seems to bear a single forward-inward-curved-tooth. Claws are visible at the end of locomotory appendages. The posterior trunk seems to be missing. The specimen has a length of about 2.2 mm.
(10) Specimen 256 (BuB 19) is accessible in the dorsal (Figure 5D) and ventral view (Figure 5E,F). Each stylet seems to bear a single slightly forward-inward-curved tooth (Figure 5G). Four protrusions are visible on the anterior rim of the head (Figure 5G). The specimen has a length of about 5.6 mm.
(11) Specimen 257 (BuB 29) is accessible in the ventral and dorsal view (Figure 4C,D) but largely concealed by dirt particles and cloudiness in the ventral and dorsal view. Each stylet seems to bear one forward-inward curved tooth. The specimen has a length of about 5.1 mm.
(12) Specimen 258 (BuB 12) is well accessible in the dorsal and ventral view (Figure 6A,B,D). Each stylet seems to bear one rather straight tooth (Figure 6C). Four protrusions are visible on the anterior rim of the head (Figure 6C). A rather slender neck seems to be apparent. Protrusions of the trunk (possible scoli) seem to be present. The specimen has a length of about 7.8 mm.
(13) Specimen 260 (PED 2770) is accessible in the ventral or dorsal view (Figure 7A,B) but partly concealed by dirt particles and cloudiness. Each stylet seems to bear one slightly forward-inward curved tooth. Claws are visible at the end of locomotory appendages. The specimen has a length of about 3.4 mm.

### 3.2. Shape Analysis

*Head and stylets:* In the analysis of the head and stylets, 44 different specimens could be included. The shape analysis of the head capsule with stylets resulted in six effective principal components, summarizing a total of 91.6% of the overall variation in the data set. The first two principal components sum up to 71.7% of the overall variation in the data set (details are located in Supplementary File S1). The principal component (PC1) explains 62.3% of the overall variation. It is dominated by the shape of the head capsule and the position of the stylets on the head. It describes rectangular to round head capsules with medially to far laterally positioned stylets, as well as the position of the single tooth in the stylets seems to influence this PC. Negative values indicate a rectangular head capsule and a more lateral position of the stylets with a more proximal position of the tooth. Positive values indicate a rather round head capsule with a concave posterior rim and a more median position of the stylets with a more distal position of the tooth.

Principal component 2 (PC2) explains 9.4% of the overall variation. It is dominated by the shape of the head capsule and the curvature of the stylets. Negative values indicate a rectangular head shape and a strongly curved proximal region of the stylet, while positive values indicate a round head shape and a straight proximal region of the stylet, slightly curved distally.

Principal component 3 (PC3) explains 7.4% of the overall variation. It is dominated by the shape of the head capsule and the length of the stylets. It describes round to rectangular head shapes and long to rather short stylets, yet the shape of the single tooth in the stylets also seems to influence this PC. Negative values indicate a round head capsule with a straight lateral rim and long stylets with curved stylet tips and a very short single tooth. Positive values indicate a rectangular head capsule and rather short stylets with a slender single tooth.

Principal component 4 (PC4) explains 6.1% of the overall variation. It is dominated by the length of the head and the width of the distal region of the stylets. Negative values indicate a rather short head with a concave posterior rim and slender stylets in the distal region, while positive values indicate a longer head with a convex posterior rim and stylets that are in the distal region as broad as in the proximal region.

Principal component 5 (PC5) explains 4.4% of the overall variation. It is dominated by the shape of stylets and the teeth, and the shape of the posterior region of the head seems to influence this PC. Negative values indicate a rectangular posterior region of the head and tapering stylets with a straight, broad single tooth, while positive values indicate a round posterior region of the head and stylets that are in the distal region as broad as in the proximal region with one longer and one shorter rather tapering tooth.

Principal component 6 (PC6) explains 2.0% of the overall variation. It is dominated by the length of the stylets and the length of the tooth in the stylets, and the shape of the posterior rim of the head also seems to influence this PC. Negative values indicate a round posterior rim of the head and slender stylets with a rather long tapering single tooth, while positive values indicate a straight posterior rim of the head and rather small distally slender stylets and a broad short single tooth.

*Stylets:* In the analysis of the stylets, 48 different specimens could be included. The shape analysis of the stylets resulted in seven effective principal components, summarizing a total of 95.1% of the overall variation in the data set. The first two principal components sum up to 69.3% of the overall variation in the data set (details are located in Supplementary File S2). The principal component (PC1) explains 44.5% of the overall variation. It is dominated by the curvature and width of the stylets and the position of the single tooth in the stylets. Negative values indicate broad stylets with strongly curved stylet tips with a more distal position of the slender single tooth, while positive values indicate a narrow stylet at the position of the single tooth with slender stylet tips and a more proximal position of the wide single tooth.

Principal component 2 (PC2) explains 24.9% of the overall variation. It is dominated by the shape of the stylet tips and the shape of the teeth. It describes broad to slender stylet tips with distinct to indistinct teeth, and the width of the proximal region of the stylets seems to influence this PC. Negative values indicate a broad proximal region of the stylets with broad rather straight stylet tips and a distinct rather long single tooth. Positive values indicate a slender proximal region of the stylets with slender stylet tips and indistinct wide teeth.

Principal component 3 (PC3) explains 12.1% of the overall variation. It is dominated by the shape of the single tooth in the stylets and the curvature of the stylet tips. Negative values indicate a wide tapering single tooth and strongly curved slender stylet tips, while positive values indicate a wide blunt single tooth and more straight slender stylet tips.

Principal component 4 (PC4) explains 6.1% of the overall variation. It is dominated by the shape of the single tooth in the stylets and the shape of the stylet tips. Negative values indicate a long tapering single tooth and slender stylet tips, while positive values indicate broad stylet tips and a wide small single tooth.

Principal component 5 (PC5) explains 3.7% of the overall variation. It is dominated by the width of the single tooth in the stylets. Negative values indicate a broad, tapering, straight single tooth, while positive values indicate a small, stout, curved single tooth.

Principal component 6 (PC6) explains 2.0% of the overall variation. It seems to be dominated by similar patterns as PC5. Negative values indicate a broad, tapering straight single tooth, while positive values indicate an indistinct broad single tooth.

Principal component 7 (PC7) explains 1.9% of the overall variation. It is dominated by the curvature of the proximal region of the stylets, and the shape of the single tooth in the stylets also seems to influence this PC. Negative values indicate a wide, strongly curved proximal region of the stylets and a curved tapering single tooth, while positive values indicate a straight, rather slender proximal region of the stylets and a stout tooth.

*Head capsule:* In the analysis of the head, 48 different specimens could be included. The shape analysis of the head capsule resulted in six effective principal components, summarizing a total of 90.3% of the overall variation in the data set. The first two principal components sum up to 62.1% of the overall variation in the data set (details are located in Supplementary File S3). Principal component 1 (PC1) explains 43.8% of the overall variation. It is dominated by the width of the head and the shape of the posterior rim. Negative values indicate a slender head with a straight posterior rim, while positive values indicate a wide head with a concave posterior rim.

Principal component 2 (PC2) explains 18.3% of the overall variation. It is dominated by the shape of the anterior edge of the head and the shape of the posterior rim of the head. Negative values indicate an anterior edge with a median projection and a rather concave posterior rim of the head, while positive values indicate a rather straight anterior edge of the head and a convex posterior region.

Principal component 3 (PC3) explains 12.8% of the overall variation. It appears to be dominated by similar patterns as principal component 2 (PC2). Negative values indicate an anterior rim of the head with a broad median protrusion and a convex posterior region laterally drawn out. Positive values indicate an anterior rim of the head with a narrow median protrusion, laterally drawn out, and a concave posterior rim of the head.

Principal component 4 (PC4) explains 7.7% of the overall variation. It is dominated by the width of the head and the shape of the lateral rim of the head. Negative values indicate a wide head and a convex lateral rim, while positive values indicate a slender head and a concave lateral rim.

Principal component 5 (PC5) explains 5.0% of the overall variation. It is dominated by the shape of the anterior edge and the posterior rim of the head. Negative values indicate an anterior rim of the head with a narrow median protrusion and a convex posterior rim of the head, while positive values indicate a rather straight anterior edge and a concave posterior rim of the head.

Principal component 6 (PC6) explains 2.8% of the overall variation. It is dominated by the shape of the anterior region of the head. Negative values indicate a distinct tapering anterior region of the head, while positive values indicate an indistinct rounded anterior region of the head.

*Entire body including stylets:* In the analysis of the entire body with stylets, 25 different specimens could be included. The shape analysis of the body outline, including the head capsule and stylets, resulted in seven effective principal components, summarizing a total of 94.3% of the overall variation in the data set. The first two principal components sum up to 80.2% of the overall variation in the data set (details are located in Supplementary File S4). Principal component 1 (PC1) explains 64.5% of the overall variation. It is dominated by the width of the head and the shape of the stylets. It describes wide to small heads with long broad to short slender stylets, and the shape of the posterior region of the body seems to influence this PC. Negative values indicate wide heads with a fluent transition between head and trunk and a rather straight, slender proximal region of the stylets with broad stylet tips and a rounded posterior region of the body. Positive values indicate slender heads with a clear distinction from the trunk and a broad proximal region of the stylets with narrow stylet tips and a long narrow posterior region of the body.

Principal component 2 (PC2) explains 15.7% of the overall variation. It is dominated by the shape of the head capsule and the stylets, and the shape of the posterior rim of the body seems to influence this PC. Negative values indicate a slender head capsule with a clear distinction from the trunk with strongly curved tapering stylets and a convex posterior rim, while positive values indicate an even more slender head capsule with a fluent transition between head and trunk with straight narrow stylets and a posterior rim with a narrow median protrusion.

Principal component 3 (PC3) explains 4.5% of the overall variation. It is dominated by the shape of the lateral rim of the trunk and the shape of the single tooth in the stylets. Negative values indicate a strongly convex lateral rim of the trunk and a distinct single tooth in the stylets, while positive values indicate a rather straight lateral rim of the trunk and indistinct tooth in the stylets.

Principal component 4 (PC4) explains 3.4% of the overall variation. It is dominated by the shape of the posterior region of the body and the width of the stylet tips. Negative values indicate a far lateral, slightly tapering posterior region of the body and narrow stylet tips, while positive values indicate a strongly medial tapering posterior region of the body and broad stylet tips.

Principal component 5 (PC5) explains 2.3% of the overall variation. It is dominated by the curvature of the stylets and the width of the posterior region of the body. Negative values indicate a slender tapering posterior region of the body and short straight stylets, while positive values indicate a wide, less tapering posterior rim of the body and long, strongly curved stylets.

Principal component 6 (PC6) explains 2.1% of the overall variation. It is dominated by the posterior rim of the body and the curvature of the stylets. Negative values indicate a posterior rim of the body with a narrow median protrusion and straight narrow stylets, while positive values indicate a straight posterior rim of the body and strongly curved tapering stylets.

Principal component 7 (PC7) explains 1.8% of the overall variation. It appears to be dominated by similar patterns as PC6. Negative values indicate a posterior rim of the body with a narrow median protrusion and rather straight stylets, while positive values indicate a rounded posterior rim of the body and strongly curved stylets.

*Entire body without stylets:* In the analysis of the body without stylets, 49 different specimens could be included. The shape analysis of the body outline, including the head without stylets, resulted in seven effective principal components, summarizing a total of 94.8% of the overall variation in the data set. The first two principal components sum up to 71.6% of the overall variation in the data set (details are located in Supplementary File S5). Principal component 1 (PC1) explains 49.7% of the overall variation. It is dominated by the shape of the head. It describes rectangular to triangular head shapes, and the curvature of the posterior rim of the body seems to influence this PC. Negative values indicate a slightly convex curved lateral rim of the trunk and a rectangular head shape with a fluent transition between head and trunk, while positive values indicate a strongly convex curved lateral rim of the trunk and a triangular head shape with a clear distinction from the trunk.

Principal component 2 (PC2) explains 21.9% of the overall variation. It is dominated by the size of the head. It describes larger to smaller heads, and the shape of the posterior rim of the body seems to influence this PC. Negative values indicate a large rectangular head and a rather rounded posterior rim of the body, while positive values indicate a smaller round head and a posterior rim of the body with a narrow median protrusion.

Principal component 3 (PC3) explains 8.9% of the overall variation. It appears to be dominated by similar patterns as principal component 2 (PC2). Negative values indicate a large rectangular head and a rounded posterior rim of the body, while positive values indicate a smaller round head and a posterior rim of the body with a narrow, elongated posterior region.

Principal component 4 (PC4) explains 6.2% of the overall variation. It is dominated by the size of the head and the shape of the posterior region of the body. Negative values indicate a smaller rounded head and a concave posterior rim of the body, while positive values indicate a larger rectangular head and a posterior rim of the body with a narrow, elongated posterior region.

Principal component 5 (PC5) explains 4.2% of the overall variation. It is dominated by the size of the head capsule and the length of the posterior region of the body. Negative values indicate a smaller head capsule and an elongated tapering posterior region of the body, while positive values indicate a larger head capsule and a short and tapering posterior region of the body.

Principal component 6 (PC6) explains 2.1% of the overall variation. It is dominated by the shape of the head capsule and the shape of the posterior region of the body. Negative values indicate a round head shape and a tapering posterior region, while positive values indicate an elongated rectangular head shape and a rounded posterior region.

Principal component 7 (PC7) explains 1.8% of the overall variation. It is dominated by similar patterns as PC6. Negative values indicate an elongated rounded head and a rounded posterior region of the body, while positive values indicate an elongated rectangular head and a tapering posterior region of the body.

*Head, stylets, and prothorax:* In the analysis of the head, prothorax, and stylets, 25 different specimens could be included. The shape analysis of the head with stylets and prothorax resulted in eight effective principal components, summarizing a total of 93.5% of the overall variation in the data set. The first two principal components sum up to 64.2% of the overall variation in the data set (details are located in Supplementary File S6). Principal component 1 (PC1) explains 52.3% of the overall variation. It is dominated by the shape of the lateral rim of the head, the position of the single tooth in the stylets, and the shape of the posterior region of the prothorax. It describes straight to convex lateral rims of the head, proximally to distally located teeth, and straight to tapering posterior regions of the prothorax, and the shape and the position of the stylets on the head seem to influence this PC. Negative values indicate an elongated head with a straight lateral rim and far laterally positioned stylets on the head and a proximal position of a wide, single tooth in rather straight stylets. Positive values indicate a wide head with a convex lateral rim and far medially positioned stylets on the head with a distal position of a narrow single tooth in strongly curved stylets.

Principal component 2 (PC2) explains 12.0% of the overall variation. It is dominated by the shape of the stylets and the shape of the prothorax. Negative values indicate a rather short prothorax with a convex lateral rim and strongly curved stylets that are in the distal region as broad as in the proximal region, while positive values indicate an elongated rather round prothorax and less curved tapering stylets.

Principal component 3 (PC3) explains 8.4% of the overall variation. It is dominated by the shape of the prothorax and the shape of the stylets. Negative values indicate long, rather straight stylets and a rather short round prothorax, while positive values indicate short, more curved stylets and an elongated posteriorly tapering prothorax.

Principal component 4 (PC4) explains 7.1% of the overall variation. It is dominated by the shape of the single tooth in the stylets and the shape of the prothorax. Negative values indicate a narrow single tooth in the stylets and a rather small prothorax with a fluent transition between head and prothorax, while positive values indicate a wide tapering single tooth in the stylets and a rather broad prothorax with a clear distinction from the head.

Principal component 5 (PC5) explains 4.8% of the overall variation. It is dominated by the shape of the stylet tips and the shape of the prothorax. Negative values indicate long curved stylet tips and an elongated prothorax with a fluent transition between head and prothorax, while positive values indicate short, rather straight stylet tips and a short, broad prothorax with a clear distinction from the head.

Principal component 6 (PC6) explains 4.3% of the overall variation. It is dominated by the shape of the single tooth in the stylets and the shape of the prothorax. Negative values indicate a wide tapering single tooth in the stylets and a rather short rectangular prothorax, while positive values indicate a single rounded tooth and a round, elongated prothorax.

Principal component 7 (PC7) explains 2.7% of the overall variation. It is dominated by the shape of the head capsule and the shape of the single tooth in the stylets. Negative values indicate a round head shape and a wide tapering single tooth in the stylets, while positive values indicate a rather rectangular head shape and a rather slender rounded tooth in the stylets.

Principal component 8 (PC8) explains 2.0% of the overall variation. It is dominated by the shape of the single tooth in the stylets, and the shape of the head also seems to influence this PC. Negative values indicate a round head with a wide single tooth in the stylets, while positive values indicate a rectangular head with a rather narrow tapering single tooth in the stylets.

*Head and prothorax (no stylets):* In the analysis of the head and prothorax, 29 different specimens could be included. The shape analysis of the head capsule without stylets but with prothorax resulted in six effective principal components, summarizing a total of 92.7% of the overall variation in the data set. The first two principal components sum up to 73.2% of the overall variation in the data set (details are located in Supplementary File S7). Principal component 1 (PC1) explains 48.5% of the overall variation. It is dominated by the shape of the prothorax. Negative values indicate a slender prothorax with a fluent transition between the head and prothorax, while positive values indicate a wide prothorax with a clear distinction from the head.

Principal component 2 (PC2) explains 24.7% of the overall variation. It is dominated by the relative lengths of the head and prothorax. Negative values indicate a short, broad head with an elongated prothorax, while positive values indicate a short, broad prothorax with an elongated head.

Principal component 3 (PC3) explains 9.8% of the overall variation. It is dominated by the shape of the lateral rim of the head and the shape of the posterior rim of the prothorax. Negative values indicate a rather straight posterior rim of the prothorax and a straight lateral rim of the head, while positive values indicate a convex posterior rim of the prothorax and a convex lateral rim of the head.

Principal component 4 (PC4) explains 5.0% of the overall variation. It is dominated by the position of the widest part of the head. Negative values indicate a further posteriorly located widest part of the head, while positive values indicate a further anteriorly located widest part of the head.

Principal component 5 (PC5) explains 2.4% of the overall variation. It seems to be dominated by similar patterns as PC3. Negative values indicate a straight lateral rim of the head and a convex posterior rim of the prothorax, while positive values indicate a convex lateral rim of the head and a rather straight posterior rim of the prothorax.

Principal component 6 (PC6) explains 2.3% of the overall variation. It seems to be dominated by similar patterns as PC3 and PC5. Negative values indicate a rather straight posterior rim of the prothorax and a convex lateral rim of the head, while positive values indicate a convex posterior rim of the prothorax and a straight lateral rim of the head.

*Prothorax:* In the analysis of the prothorax, 28 different specimens could be included. The shape analysis of the prothorax resulted in six effective principal components, summarizing a total of 98.0% of the overall variation in the data set. The first two principal components sum up to 87.8% of the overall variation of the data set (details are located in Supplementary File S8). Principal component 1 (PC1) explains 74.9% of the overall variation. It is dominated by the width of the prothorax. Negative values indicate a slender anterior region of the prothorax and a rather wide posterior region. Positive values indicate a short and wide prothorax.

Principal component 2 (PC2) explains 12.8% of the overall variation. It is dominated by the location of the widest part of the prothorax. Negative values indicate a further anteriorly located widest part of the prothorax, while positive values indicate a further posteriorly located widest part of the prothorax.

Principal component 3 (PC3) explains 5.1% of the overall variation. It is dominated by the shape of the prothorax. Negative values indicate a rectangular prothorax with a laterally drawn-out posterior part, while positive values indicate a wide triangular posterior region of the prothorax and a slender rectangular anterior region.

Principal component 4 (PC4) explains 3.1% of the overall variation. It seems to be dominated by similar patterns as principal component 2 (PC2). Negative values indicate a further anteriorly located widest part of the prothorax, while positive values indicate a further posteriorly located widest part of the prothorax.

Principal component 5 (PC5) explains 1.2% of the overall variation. It seems to be dominated by similar patterns as PC2 and PC4. Negative values indicate a further anteriorly located widest part of the prothorax, while positive values indicate a further posteriorly located widest part of the prothorax.

Principal component 6 (PC6) explains 0.8% of the overall variation. It is dominated by the shape of the prothorax. Negative values indicate a laterally drawn-out posterior part of the prothorax, while positive values indicate a round prothorax shape.

*Body without head capsule, i.e., trunk:* In the analysis of the trunk, 29 different specimens could be included. The shape analysis of the trunk resulted in seven effective principal components, summarizing a total of 94.8% of the overall variation in the data set. The first two principal components sum up to 64.7% of the overall variation in the data set (details are located in Supplementary File S9). Principal component 1 (PC1) explains 44.1% of the overall variation. It is dominated by the curvature of the lateral rim of the trunk and the shape of the posterior region. It describes strongly curved to less curved lateral rims of the trunk and elongated tapering posterior regions to rounded posterior regions, and the shape of the anterior rim of the trunk seems to influence this PC. Negative values indicate a strongly curved, rather wide trunk with a lateral indentation at the anterior rim and an elongated posterior region, while positive values indicate a slightly curved, rather slender trunk with a rounded posterior and anterior region.

Principal component 2 (PC2) explains 20.6% of the overall variation. It is dominated by the shape of the anterior and posterior regions of the trunk. Negative values indicate an elongated rounded anterior region and an elongated tapering posterior region, while positive values indicate a straight anterior rim and a slightly tapering posterior region.

Principal component 3 (PC3) explains 12.2% of the overall variation. It is dominated by the shape of the anterior region of the trunk and the length of the trunk end. Negative values indicate a rectangular anterior region of the trunk and an elongated trunk end, while positive values indicate a slender round anterior region and a short trunk end.

Principal component 4 (PC4) explains 9.1% of the overall variation. It appears to be dominated by similar patterns as PC3. Negative values indicate a rectangular anterior region and a short trunk end, while positive values indicate a slender round anterior region and an elongated trunk end.

Principal component 5 (PC5) explains 4.1% of the overall variation. It is dominated by the shape of the lateral anterior region of the trunk and the width of the trunk end. Negative values indicate a lateral indentation in the anterior region and a slender trunk end, while positive values indicate a rounded lateral anterior region and a broad trunk end.

Principal component 6 (PC6) explains 2.7% of the overall variation. It appears to be dominated by similar patterns as PC3 and PC4. Negative values indicate a rounded slender anterior region and a broad, tapering trunk end, while positive values indicate a rounded, broad anterior region and an elongated tapering trunk end.

Principal component 7 (PC7) explains 1.8% of the overall variation. It appears to be dominated by similar patterns as PC3, PC4, and PC6. Negative values indicate a short tapering anterior region of the trunk and an elongated tapering trunk end, while positive values indicate a rounded anterior region and a straight trunk end.

*Trunk without prothorax:* In the analysis of the trunk without the prothorax, 29 different specimens could be included. The shape analysis of the trunk without the prothorax resulted in seven effective principal components, summarizing a total of 93.7% of the overall variation in the data set. The first two principal components sum up to 62.4% of the overall variation in the data set (details are located in Supplementary File S10). Principal component 1 (PC1) explains 42.4% of the overall variation. It is dominated by the curvature of the lateral rim of the trunk and the shape of the trunk end. Negative values indicate a slightly convex lateral rim of the trunk and a rather wide tapering trunk end, while positive values indicate a strongly convex lateral rim of the trunk and a narrow tapering trunk end.

Principal component 2 (PC2) explains 20.0% of the overall variation. It is dominated by the length of the trunk end. It describes elongated to short trunk ends, and the shape of the anterior region of the trunk seems to influence this PC. Negative values indicate a strongly elongated wide trunk end and a clear distinction between the posterior thorax and the remaining trunk. Positive values indicate a short, narrow trunk end and a fluent transition between the posterior thorax and the remaining trunk.

Principal component 3 (PC3) explains 12.5% of the overall variation. It appears to be dominated by the shape of the anterior region of the trunk and the shape of the trunk end. Negative values indicate a rather tapering anterior region and a narrow, tapering, elongated trunk end, while positive values indicate a rounded anterior region and a wide, tapering, elongated trunk end.

Principal component 4 (PC4) explains 8.3% of the overall variation. It appears to be dominated by the shape of the anterior rim of the trunk and the length of the trunk end. Negative values indicate a convex anterior rim and no distinct trunk end, while positive values indicate a concave anterior rim and a distinct tapering trunk end.

Principal component 5 (PC5) explains 4.9% of the overall variation. It is dominated by the shape of the anterior region of the trunk and the width of the trunk end. Negative values indicate a rectangular anterior region of the trunk and a wide trunk end, while positive values indicate a rounded anterior region of the trunk and a narrow trunk end.

Principal component 6 (PC6) explains 3.2% of the overall variation. It is dominated by the shape of the trunk end. Negative values indicate a tapering narrow trunk end, while positive values indicate a wide trunk end.

Principal component 7 (PC7) explains 2.6% of the overall variation. It appears to be dominated by similar patterns as PC4. Negative values indicate a convex anterior rim of the trunk and a slightly elongated trunk end, while positive values indicate a concave anterior rim of the trunk and a short trunk end.

## 4. Discussion

### 4.1. Identity of the Specimens

Larvae of the group Nymphidae are generally easy to identify. The most distinct characteristic of extant and fossil specimens is the presence of a prominent single tooth in the stylets. The number of teeth can vary in specimens of the group of owllions (formerly known as “Myrmeleontidae + Ascalaphidae”, but the situation has become complicated; a simple neutral term for the group, including its assembled stem-lineage sensu Ax [95] would be Eumyrmeleontiformia; for challenges of all terminologies concerning the “stem”, see Donoghue [96]). Yet, extant larvae of Nymphidae consistently possess only one tooth. According to MacLeod [43], some larvae of Nymphidae, particularly in fossils, may have more than one tooth, and larvae of other groups may possess only one tooth. However, the general morphology of the larvae in this study, such as the relatively wide head capsule, is consistent with the interpretation of the larvae as representatives of Nymphidae, or at least as representatives of the lineage toward them (assembled stem-lineage sensu Ax [95] p. 43).

Modern owllion larvae can be identified by the organization of the walking appendages: they possess a continuous tibio-tarsus [43]. In larvae of Nymphidae, the tarsus is set off from the tibia, congruent with the herein-presented specimens [70].

Some of the new larvae also possess an additional smaller tooth (Figure 1 and Figure 3; also already demonstrated by Haug et al. [70]). While this smaller tooth does not occur in extant specimens, one or even up to three additional smaller teeth have been reported in fossil larvae (MacLeod [43] Figure 6 and Figure 7). Another characteristic of certain fossil specimens is the presence of (mostly) four prominent forward-oriented spines or strong setae (Figure 3, Figure 5, and Figure 6) arising from the labrum region of the head capsule. This characteristic is particularly evident in some Cretaceous larvae that were formally described as the species *Nymphavus progenitor* [68]. Larvae with such protrusions on the head either represent this species or are at least a species closely related to it [70]. Some of these spine-bearing specimens also have a smaller additional tooth [75]; as *Nymphavus progenitor* was firmly resolved as a representative of Nymphidae [68], the specimens with the additional smaller tooth can be interpreted as representatives of Nymphidae as well. As some of the fossils still retain a plesiomorphic empodium [75], they may more likely be derivatives of the assembled stem lineage (this entire group, total group sensu Jefferies [97], could be referred to as Nymphiformia).

### 4.2. Differences in Shape Aspects

**Figure 8:**
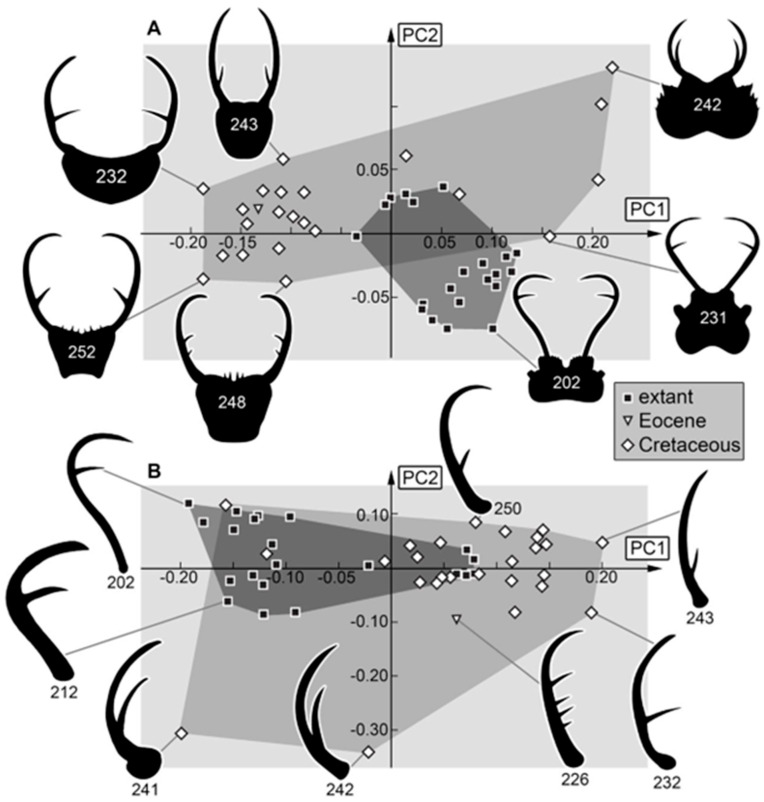
Morphospaces represented by scatter plots of the principal components (PCs) 1 and 2 of (**A**) head + stylet shapes and (**B**) stylet shapes.

The data set included multiple specimens from the Cretaceous, but only a single specimen from the Eocene period [43]. The extant fauna can, therefore, be effectively compared with the Cretaceous one but not with the Eocene one. The Eocene larva does not plot far from the other fossil larvae, though (e.g., Figure 8A), and therefore does not introduce any bias or distortions in the results. There are recognizable differences between the shapes of the Cretaceous larvae and those of the modern fauna.

*Head and stylets:* The morphospace occupation of the head and stylet shape is larger in the Cretaceous (Figure 8A). The lower left area of the morphospace seems to be occupied by Nymphavus-type larvae (e.g., specimens 248, 252; Figure 8A). They plot close to (other) fossil larvae with a lateral position of the stylets and a straight proximal stylet shape in the upper left area of the morphospace. Some outliers plot in the upper right area of the morphospace (larvae described in Haug et al. [69]). These larvae have broad heads with additional lateral protrusions and prominent stylets possessing one forward-inward curved tooth. The lower right and middle areas of the morphospace seem to be occupied by most of the extant larvae, yet they form two distinct groups (as in the earlier study by Haug et al. [70]). The larvae of the “myiodactylid” type (larvae of Myiodactylinae; ref. [90]) plot in the lower right. They have wide heads with straight or even slightly outward curved stylets that are distally sharply curved inward and have a further distally located single tooth (e.g., specimen 202, 209, Figure 8A; ref. [70]). It is remarkable that no fossil specimen plots inside the area occupied by extant “myiodactylid”-type specimens. The “myrmeleontid” type (larvae of Nymphinae; ref. [90]) possess rather square-shaped heads and simply curved stylets with the single tooth sitting rather in the middle of the stylet [70] and plot in the middle of the morphospace as they do not have very distinct features. Overall, it seems that larvae of the Cretaceous were more diverse in their morphology concerning their head and stylet. The “myrmeleontid” type seems to have already existed during the Cretaceous. The “myiodactylid” type larvae might have evolved only later. However, it cannot be excluded that the “myiodactylid”-type larvae were already present in the Cretaceous but did not get trapped in amber, for example, due to different habitat preferences.

*Stylets:* The shape analysis of the stylets shows similar outcomes, but with an even larger Cretaceous occupation of the morphospace (Figure 8B). This pattern indicates the loss of certain stylet types over time and, therefore, most likely a loss of certain ecological roles. Only the extreme forms of two “myiodactylid”-type larvae (202, 209) plot outside the morphospace occupied by specimens of the Cretaceous, which could support that at least some “myiodactylid”-type larvae evolved only after the Cretaceous. The two extreme fossils, 241 and 242, plot outside modern specimens and far away from other fossil specimens [69]. They seem to have had special stylets coupled to a specific feeding style that is not represented in the modern fauna. Also remarkable is that if 241 and 242 were not included, the Eocene specimen would plot outside the Cretaceous fossils. This indicates a special form of the stylet in the Eocene that may not have existed during the Cretaceous or the extant fauna.

**Figure 9:**
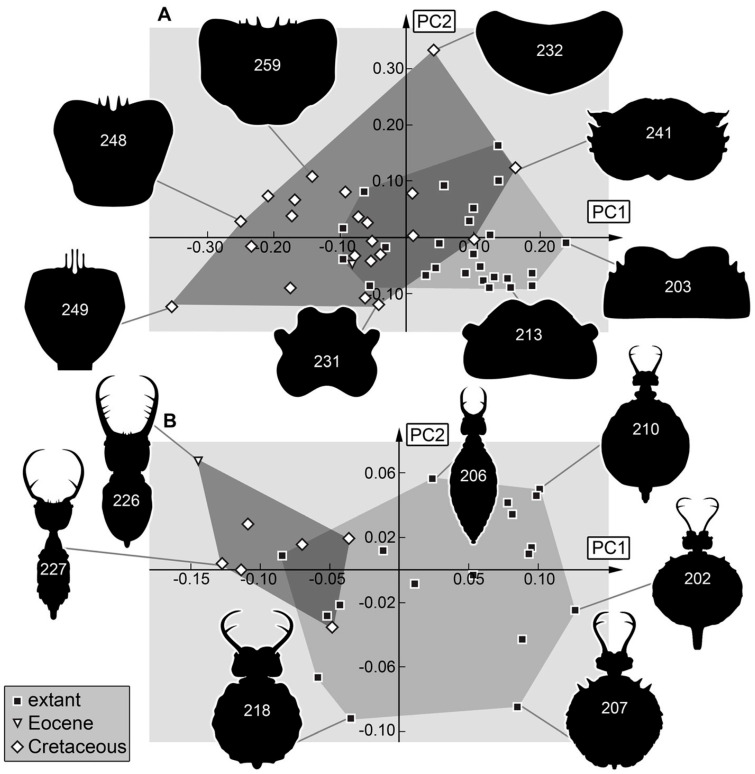
Morphospaces represented by scatter plots of the principal components (PCs) 1 and 2 of (**A**) head capsule shapes and (**B**) entire body + stylet shapes.

*Head capsule:* The Cretaceous still occupies a larger area of the morphospace than the modern fauna (Figure 9A). Again, a separation into two groups of the extant specimens is recognizable. Five specimens plot clearly inside the morphospace occupied by the fossils (with a negative value for PC1). These five specimens are plotted already in the fossil morphospace for the analyses of the head and the stylet (Figure 8A). This is apparent in the head + stylet shape, and the stylet shape supports that the lineage with “myrmeleontid”-type larvae was already present in the Cretaceous. It is important to recognize that the fossil morphospace is only extended into the modern-day “myiodactylid” type by the two special specimens 241 and 242 [69]. While most fossil larvae plot on the rather left side of the morphospace, these two specimens plot deep inside the modern larvae. They have a similar head shape as larvae of the modern “myiodactylid” type. Still, due to the differences in the stylets, they must have had a feeding ecology not present today. The Nymphavus-type larvae occupy a large area of the morphospace and extend to the left side (e.g., specimens 248, 249, Figure 9A). This also indicates a difference in morphology and ecology between the Cretaceous and the modern larvae. In general, the shape of the head capsule has largely shifted from an area occupied in the Cretaceous to a new area occupied in the modern-day fauna.

**Figure 10:**
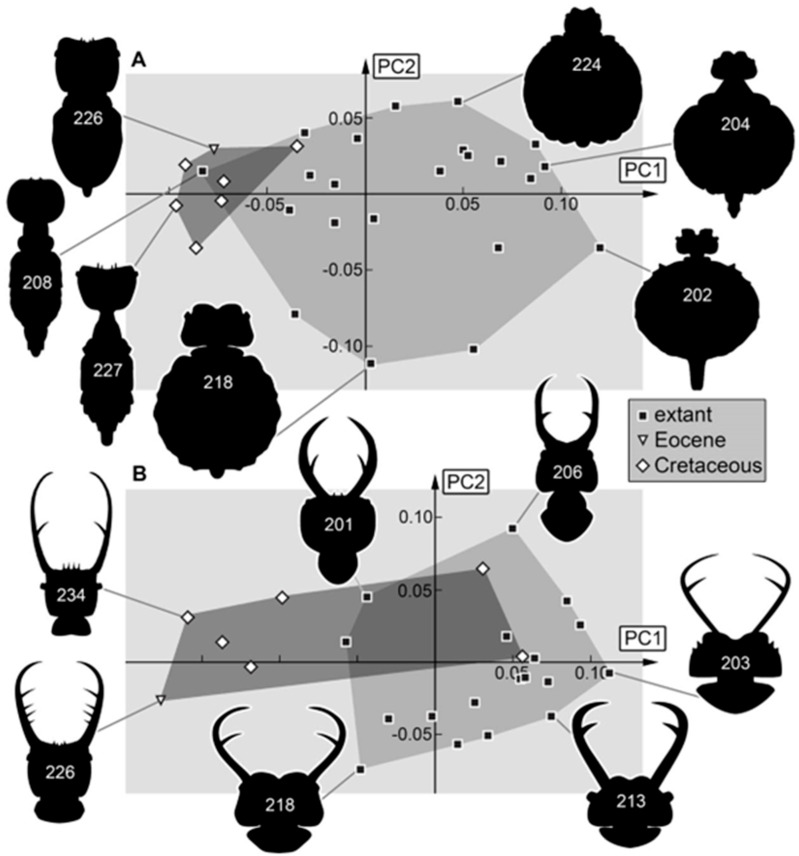
Morphospaces represented by scatter plots of the principal components (PCs) 1 and 2 of (**A**) body shapes without stylets and (**B**) head + stylet + prothorax shapes.

*Entire body:* Body shapes with and without stylets both seem to have a similar distribution in the morphospace, with the occupied area of the modern fauna being much larger (Figure 9B and Figure 10A). It must be noted that the data set for the Cretaceous is much smaller due to poor preservation of this body region in many amber fossils. There is probably also a sample bias here. Most modern larvae in the literature are stage 3 individuals, while most of the fossils most likely represent stage 1 specimens.

The fossils seem to have had larger heads compared to the trunk than the extant specimens (again indicating a younger developmental stage). Only extant specimen 208 plots clearly inside the morphospace occupied by the fossils (for both analyses). This indicates that the forms with broader heads compared to the trunk are still present in the modern fauna and further supports that these are the younger larvae. Similar patterns could be seen in a comparable analysis of long-nosed antlions, i.e., larvae of silky lacewings (Psychopsidae; ref. [73]), but not in larvae of dragon lacewings [74]. The phenomenon might therefore be restricted to the groups within Myrmeleontiformia.

Besides the possible sample bias, a certain evolutionary signal can be detected as well. Myrmeleontiformian larvae had more slender appearances in the Cretaceous [67,68], and this also applies to the herein-presented larvae. What is further remarkable is the extreme form of the single Eocene specimen. This might be caused by the stylets, as the position of the Eocene specimen in the morphospace of the body without a stylet (Figure 10A) is not as extreme as it is for the body with a stylet (Figure 9B).

**Figure 11:**
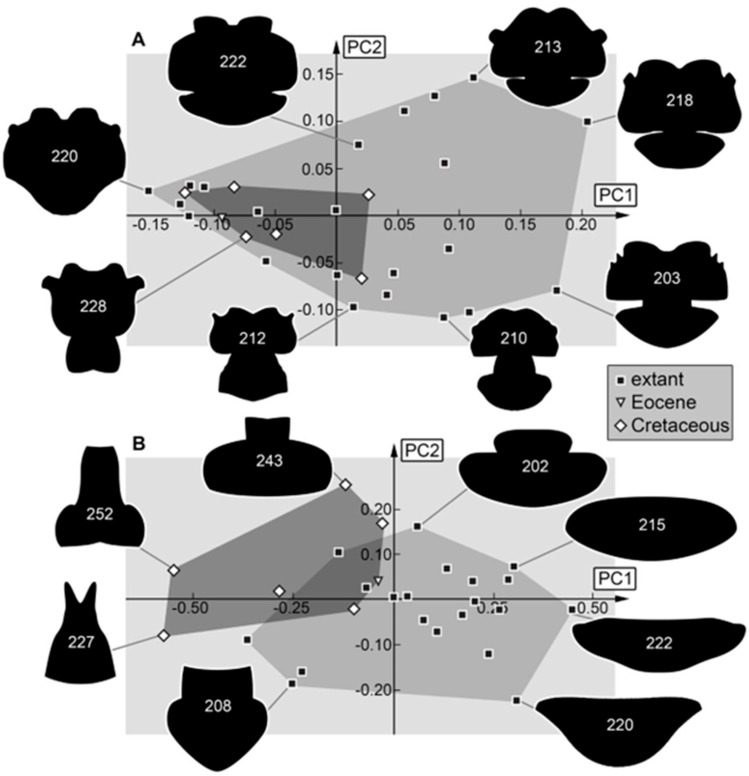
Morphospaces represented by scatter plots of the principal components (PCs) 1 and 2 of (**A**) prothorax + head shapes without stylets and (**B**) prothorax shapes.

*Prothorax:* The prothorax is not a distinct characteristic of larvae of Nymphidae. It can be noticed that larvae with a wide head often have a wider prothorax, and larvae with rather slender or rectangular heads have a rather elongated slender prothorax (Figure 10B and Figure 11A,B). In the analysis of the prothorax, some fossils plot outside the morphospace occupied by the extant ones (Figure 11B). These forms have a longer anterior end (so possibly a longer cervix?) than the extant ones. The extant ones also show forms without an elongated anterior end that are not present in the fossil forms. If the head and the prothorax are used, the anterior elongation of the prothorax does not influence the shape as for only prothorax. Furthermore, all fossil forms seem present in the modern fauna (Figure 11A). If also the stylets are concerned, only two fossils plot inside the morphospace occupied by the extant specimens (Figure 10B). This pattern again emphasizes the difference in the mouthparts, indicating a difference in the feeding ecology between the fossil larvae and those of the modern fauna. The Eocene specimen plots far outside the morphospace of the Cretaceous and extant ones, while for the head + prothorax (Figure 11A) and only prothorax (Figure 11B), the Eocene specimen plots inside or near the morphospace occupied by the Cretaceous and extant specimens. Again, the special shape of the stylet of the Eocene specimen seems to be the reason for its position.

**Figure 12:**
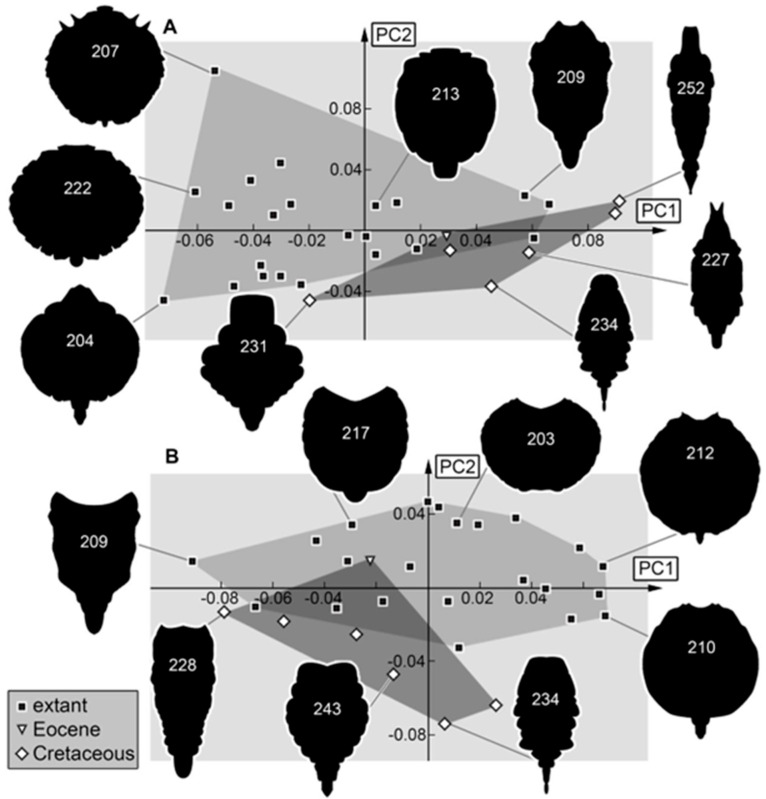
Morphospaces represented by scatter plots of the principal components (PCs) 1 and 2 of (**A**) trunk shapes and (**B**) trunk shapes without prothorax.

*Trunk:* In the trunk shapes (with and without prothorax), the Cretaceous larvae occupy an area different from the modern fauna (Figure 12A,B). The area occupied by the extant specimens is also larger than the area occupied by the fossils. These results are similar to those of the analysis of the entire body and seem therefore affected by differences in ontogenetic stages and the change to broader trunks.

Similar to an earlier analysis in larvae of silky lacewings [73], the biggest differences between the Cretaceous and modern fauna are recognized in the head and the stylets. When including more aspects of the trunk, the modern fauna seems to show more variability, which is likely related to the fact that amber has a higher percentage of smaller larvae than the extant fauna. It can be assumed that the differences in head and stylet shape are strongly correlated to aspects of the functional morphology of feeding. The observed differences in the trunk are, however, likely coupled to differences in the ontogenetic status of the larvae and are more challenging to interpret.

### 4.3. Differences to Earlier Studies

**Figure 13:**
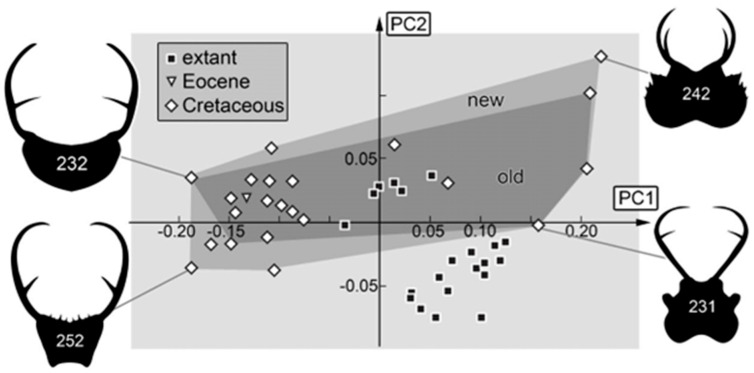
Scatterplot of PC1 vs. PC2 values. Saturation plot comparing new fossil data with previous data set from Haug et al. [70]. The polygon of the new data set occupies a larger area than the polygon of the old data set.

The earlier study by Haug et al. [70] already recognized that the area occupied by the morphospace of Cretaceous larvae of split-footed lacewings was larger than that of the modern ones concerning head and stylet shape. As the current study has added numerous specimens to the Cretaceous subsample, it is not surprising that the result is still a decrease in diversity. Yet, it is of interest how much the addition of new specimens influences an analysis, as presumably, at a certain point, basically all morphologies are already represented, and adding more specimens will not add more variation to an analysis. In other words, one can expect that the data set is saturated at a certain point. Comparing the occupied area of the Cretaceous larvae in the study by Haug et al. [70] with that in the current data set reveals quite some differences, indicating that saturation is not yet achieved (Figure 13). This result is similar to those of other studies (e.g., [73]), indicating that additional finds in the Cretaceous fauna are to be expected in the future.

### 4.4. Changes in Morphological Diversity and Abundance

As in earlier studies, the present analysis indicates that the morphological diversity of larval forms of Nymphidae was higher in the Cretaceous, pointing to greater ecological diversity in the past. Some of the ecological roles fulfilled by the now-extinct larvae may have been partly taken over by some larvae of owllions [71], yet others are likely not represented at all in the modern fauna. The latter may have been coupled to prey that likewise went extinct.

Another factor that becomes more and more apparent is that lacewing larvae were not only more diverse concerning their shapes (and ecologies), but they seem to have been also quite abundant. Several hundred larvae are already known, and the data set is, as also demonstrated here, still growing [58,71,73]. In comparison to other larvae (e.g., [58]), larvae of Nymphidae seem abundant, if not dominating the fauna. This abundance is not known from the modern fauna or younger amber fauna. In the latter, lacewing larvae are significantly less common, not only in absolute numbers but also in comparison to other larvae. While it remains challenging to obtain reliable estimates of abundances (due to the biased nature of reports of larvae), there are at least some indications that lacewing larvae in general, but also especially split-footed lacewing larvae, might have played a much more important role in Cretaceous faunas.

Currently, our picture of the Cretaceous faunas is still incomplete but keeps growing. For example, ground beetle larvae have been basically absent in Kachin amber, but now start to become more recognized [98,99,100]. It remains to be seen whether the possible dominance of lacewing larvae is a mere artifact or an indication of a much more drastic loss in ecological functions of lacewing larvae.

## Data Availability

All data from this study are available in this paper and the associated papers.

## Supplementary Materials

The following supporting information can be downloaded at https://www.mdpi.com/article/10.3390/insects16020125/s1: Supplementary Table S1: information on the specimens included in the analyses. Supplementary Files S1–S10: the results of the different shape analyses. Figure 1, Figure 2, Figure 3, Figure 4, Figure 5, Figure 6 and Figure 7 in high resolution are available at https://doi.org/10.5281/zenodo.13729878 (accessed on 28 December 2024).

## References

[1] Dunn R.R. (2005). Modern insect extinctions, the neglected majority. Conserv. Biol..

[2] Hallmann C.A., Sorg M., Jongejans E., Siepel H., Hofland N., Schwan H., Stenmans W., Müller A., Sumser H., Hörren T. (2017). More than 75 percent decline over 27 years in total flying insect biomass in protected areas. PLoS ONE.

[3] Hallmann C.A., Ssymank A., Sorg M., de Kroon H., Jongejans E. (2021). Insect biomass decline scaled to species diversity: General patterns derived from a hoverfly community. Proc. Natl. Acad. Sci. USA.

[4] Forister M.L., Pelton E.M., Black S.H. (2019). Declines in insect abundance and diversity: We know enough to act now. Conserv. Sci. Pract..

[5] Goulson D. (2019). The insect apocalypse, and why it matters. Curr. Biol..

[6] Almond R.E., Grooten M., Peterson T. (2020). Living Planet Report 2020—Bending the Curve of Biodiversity Loss.

[7] Van der Sluijs J.P. (2020). Insect decline, an emerging global environmental risk. Curr. Opin. Environ. Sustain..

[8] Fenoglio M.S., Calviño A., González E., Salvo A., Videla M. (2021). Urbanisation drivers and underlying mechanisms of terrestrial insect diversity loss in cities. Ecol. Entomol..

[9] Schachat S.R., Labandeira C.C. (2021). Are insects heading toward their first mass extinction? Distinguishing turnover from crises in their fossil record. Ann. Entomol. Soc. Amer..

[10] Wagner D.L., Grames E.M., Forister M.L., Berenbaum M.R., Stopak D. (2021). Insect decline in the Anthropocene: Death by a thousand cuts. Proc. Natl. Acad. Sci. USA.

[11] Weisser W., Blüthgen N., Staab M., Achury R., Müller J. (2023). Experiments are needed to quantify the main causes of insect decline. Biol. Lett..

[12] Schowalter T.D., Noriega J.A., Tscharntke T. (2018). Insect effects on ecosystem services—Introduction. Basic Appl. Ecol..

[13] Dangles O., Casas J. (2019). Ecosystem services provided by insects for achieving sustainable development goals. Ecosyst. Serv..

[14] Morimoto J. (2020). Addressing global challenges with unconventional insect ecosystem services: Why should humanity care about insect larvae?. People Nat..

[15] Mirth K.M., Riddiford L.M. (2007). Size assessment and growth control: How adult size is determined in insects. BioEssays.

[16] Zacharuk R.Y., Shields V.D. (1991). Sensilla of immature insects. Ann. Rev..

[17] Sarwar M. (2020). Typical flies: Natural history, lifestyle and diversity of Diptera. Life Cycle and Development of Diptera.

[18] Grimaldi D., Engel M.S. (2005). Evolution of the Insects.

[19] Lawrence J.F., Ślipiski A., Seago A.E., Thayer M.K., Newton A.F., Marvaldi A.E. (2011). Phylogeny of the Coleoptera based on morphological characters of adults and larvae. Ann. Zool..

[20] Yuan M.L., Zhang Q.L., Zhang L., Guo Z.L., Liu Y.J., Shen Y.Y., Shao R. (2016). High-level phylogeny of the Coleoptera inferred with mitochondrial genome sequences. Mol. Phyl. Evol..

[21] McKenna D.D., Shin S., Ahrens D., Balke M., Beza-Beza C., Clarke D.J., Donath A., Escalona H.E., Friedrich F., Letsch H. (2019). The evolution and genomic basis of beetle diversity. Proc. Natl. Acad. Sci. USA.

[22] Yeates D.K., Wiegmann B.M., Courtney G.W., Meier R., Lambkin C., Pape T. (2007). Phylogeny and systematics of Diptera: Two decades of progress and prospects. Zootaxa.

[23] Lambkin C.L., Sinclair B.J., Pape T., Courtney G.W., Skevington J.H., Meier R., Yeates D.K., Blagoderov V., Wiegmann B.M. (2013). The phylogenetic relationships among infraorders and superfamilies of Diptera based on morphological evidence. Syst. Entomol..

[24] Courtney G.W., Pape T., Skevington J.H., Sinclair B.J., Foottit R.G., Adler P.H. (2017). Biodiversity of Diptera. Insect Biodiversity: Science and Society.

[25] Timmermans M.J., Lees D.C., Simonsen T.J. (2014). Towards a mitogenomic phylogeny of Lepidoptera. Mol. Phyl. Evol..

[26] Goldstein P.Z., Foottit R.G., Adler P.H. (2017). Diversity and significance of Lepidoptera: A phylogenetic perspective. Insect Biodiversity: Science and Society.

[27] Mitter C., Davis D.R., Cummings M.P. (2017). Phylogeny and evolution of Lepidoptera. Ann. Rev. Entomol..

[28] Huber J.T., Foottit R.G., Adler P.H. (2017). Biodiversity of Hymenoptera. Insect Biodiversity: Science and Society.

[29] Peters R.S., Krogmann L., Mayer C., Donath A., Gunkel S., Meusemann K., Kozlov A., Podsiadlowski L., Petersen M., Lanfear R. (2017). Evolutionary history of the Hymenoptera. Curr. Biol..

[30] Wang M., Li L., Shih C., Gao T., Ren D., Ren D., Shih C.K., Gao T., Yao Y., Wang Y. (2019). Chapter 22: Hymenoptera—Sawflies and Wasps. Rhythms of Insect Evolution: Evidence from the Jurassic and Cretaceous in Northern China.

[31] Benefer C., Andrew P., Blackshaw R., Ellis J., Knight M. (2010). The spatial distribution of phytophagous insect larvae in grassland soils. Appl. Soil Ecol..

[32] Hynes H.B.N. (1970). The ecology of stream insects. Ann. Rev. Entomol..

[33] Hershey A.E., Lamberti G.A., Chaloner D.T., Northington R.M. (2010). Aquatic insect ecology. Ecology and Classification of North American Freshwater Invertebrates.

[34] Aspöck U., Aspöck H. (1999). Kamelhälse, Schlammfliegen, Ameisenlöwen. Wer sind sie? (Insecta: Neuropterida: Raphidioptera, Megaloptera, Neuroptera). Stapfia.

[35] Aspöck U., Aspöck H. (2007). Verbliebene Vielfalt vergangener Blüte. Zur Evolution, Phylogenie und Biodiversität der Neuropterida (Insecta: Endopterygota). Denisia.

[36] Aspöck U., Haring E., Aspöck H. (2012). The phylogeny of the Neuropterida: Long lasting and current controversies and challenges (Insecta: Endopterygota). Arthropod Syst. Phyl..

[37] Aspöck U., Plant J.D., Nemeschkal H.L. (2001). Cladistic analysis of Neuroptera and their systematic position within Neuropterida (Insecta: Holometabola: Neuropterida: Neuroptera). Syst. Entomol..

[38] Winterton S.L., Hardy N.B., Wiegmann B.M. (2010). On wings of lace: Phylogeny and Bayesian divergence time estimates of Neuropterida (Insecta) based on morphological and molecular data. Syst. Entomol..

[39] Winterton S.L., Lemmon A.R., Gillung J.P., Garzon I.J., Badano D., Bakkes D.K., Breitkreuz L.C.V., Engel M., Moriarty E.M., Liu X. (2018). Evolution of lacewings and allied orders using anchored phylogenomics (Neuroptera, Megaloptera, Raphidioptera). Syst. Entomol..

[40] Engel M.S., Winterton S.L., Breitkreuz L.C. (2018). Phylogeny and evolution of Neuropterida: Where have wings of lace taken us?. Ann. Rev. Entomol..

[41] Vasilikopoulos A., Misof B., Meusemann K., Lieberz D., Flouri T., Beutel R.G., Niehuis O., Wappler T., Rust J., Peters R.S. (2020). An integrative phylogenomic approach to elucidate the evolutionary history and divergence times of Neuropterida (Insecta: Holometabola). BMC Evol. Biol..

[42] Oswald J.D. (2018). Neuropterida Species of the World. Lacewing Digital Library, Research Publication, 1.

[43] MacLeod E.G. (1970). The Neuroptera of the Baltic Amber. I. Ascalaphidae, Nymphidae, and Psychopsidae. Psyche.

[44] Larsson S.G. (1978). Baltic Amber: A Palaeobiological Study.

[45] Grimaldi D.A., Grimaldi D.A. (2000). A diverse fauna of Neuropteroidea in amber from the Cretaceous of New Jersey. Studies on Fossils in Amber, with Particular Reference to the Cretaceous of New Jersey.

[46] Grimaldi D.A., Engel M.S., Nascimbene P.C. (2002). Fossiliferous Cretaceous amber from Myanmar (Burma): Its rediscovery, biotic diversity, and paleontological significance. Amer. Mus. Nov..

[47] Weitschat W., Wichard W. (2002). Atlas of Plants and Animals in Baltic Amber.

[48] Perrichot V. (2003). Environnements Paraliques à Ambre et à Végétaux du Crétacé Nord-Aquitain (Charentes, Sud-Ouest de la France). Ph.D. Thesis.

[49] Scheven J. (2004). Bernstein-Einschlüsse: Eine Untergegangene Welt Bezeugt die Schöpfung. Erinnerungen an die Welt vor der Sintflut.

[50] Engel M.S., Grimaldi D.A. (2007). The neuropterid fauna of Dominican and Mexican amber (Neuropterida: Megaloptera, Neuroptera). Amer. Mus. Nov..

[51] Engel M.S., Grimaldi D.A. (2008). Diverse Neuropterida in Cretaceous amber, with particular reference to the paleofauna of Myanmar (Insecta). Nova Suppl. Entomol..

[52] Wichard W., Gröhn C., Seredszus F. (2009). Aquatic Insects in Baltic Amber.

[53] Ohl M. (2011). Aboard a spider—A complex developmental strategy fossilized in amber. Naturwissenschaften.

[54] Pérez-de la Fuente R., Delclòs X., Peñalver E., Speranza M., Wierzchos J., Ascaso C., Engel M.S. (2012). Early evolution and ecology of camouflage in insects. Proc. Natl. Acad. Sci. USA.

[55] Pérez-de la Fuente R., Delclos X., Penalver E., Engel M.S. (2016). A defensive behavior and plant-insect interaction in Early Cretaceous amber–the case of the immature lacewing *Hallucinochrysa diogenesi*. Arthropod Struct. Dev..

[56] Pérez-de la Fuente R., Peñalver E., Azar D., Engel M.S. (2018). A soil-carrying lacewing larva in Early Cretaceous Lebanese amber. Sci. Rep..

[57] Pérez-de la Fuente R., Engel M.S., Azar D., Peñalver E. (2019). The hatching mechanism of 130-million-year-old insects: An association of neonates, egg shells and egg bursters in Lebanese amber. Palaeontology.

[58] Pérez-de la Fuente R., Engel M.S., Delclòs X., Peñalver E. (2020). Straight-jawed lacewing larvae (Neuroptera) from Lower Cretaceous Spanish amber, with an account on the known amber diversity of neuropterid immatures. Cretac. Res..

[59] Gröhn C. (2015). Einschlüsse im Baltischen Bernstein.

[60] Xia F., Yang G., Zhang Q., Shi G., Wang B. (2015). Amber: Life Through Time and Space.

[61] Liu H., Luo C., Jarzembowski E.A., Xiao C. (2022). *Acanthochrysa langae* gen. et sp. nov., a new lacewing larva (Neuroptera: Chrysopoidea) from mid-Cretaceous Kachin amber. Cretac. Res..

[62] Liu X., Shi G., Xia F., Lu X., Wang B., Engel M.S. (2018). Liverwort mimesis in a Cretaceous lacewing larva. Curr. Biol..

[63] Liu X., Zhang W., Winterton S.L., Breitkreuz L.C., Engel M.S. (2016). Early morphological specialization for insect-spider associations in Mesozoic lacewings. Curr. Biol..

[64] Wang B., Xia F., Engel M.S., Perrichot V., Shi G., Zhang H., Chen J., Jarzembowski E.A., Wappler T., Rust J. (2016). Debris-carrying camouflage among diverse lineages of Cretaceous insects. Sci. Adv..

[65] Wang B., Shi G., Xu C., Spicer R.A., Perrichot V., Schmidt A.R., Feldberg K., Heinrichs J., Chény C., Pang H. (2021). The mid-Miocene Zhangpu biota reveals an outstandingly rich rainforest biome in East Asia. Sci. Adv..

[66] Wichard W. (2017). Family Nevrorthidae (Insecta, Neuroptera) in mid-Cretaceous Burmese amber. Palaeodiversity.

[67] Makarkin V.N. (2018). Re-description of *Grammapsychops lebedevi* Martynova, 1954 (Neuroptera: Psychopsidae) with notes on the Late Cretaceous psychopsoids. Zootaxa.

[68] Badano D., Engel M.S., Basso A., Wang B., Cerretti P. (2018). Diverse Cretaceous larvae reveal the evolutionary and behavioural history of antlions and lacewings. Nat. Comm..

[69] Haug C., Zippel A., Hassenbach C., Haug G.T., Haug J.T. (2022). A split-footed lacewing larva from about 100-million-year-old amber indicates a now extinct hunting strategy for neuropterans. Bull. Geosci..

[70] Haug G.T., Haug C., van der Wal S., Müller P., Haug J.T. (2022). Split-footed lacewings declined over time: Indications from the morphological diversity of their antlion-like larvae. PalZ.

[71] Haug C., Braig F., Haug J.T. (2023). Quantitative analysis of lacewing larvae over more than 100 million years reveals a complex pattern of loss of morphological diversity. Sci. Rep..

[72] Luo C., Liu H., Jarzembowski E.A. (2022). High morphological disparity of neuropteran larvae during the Cretaceous revealed by a new large species. Geol. Mag..

[73] Hassenbach C., Buchner L., Haug G.T., Haug C., Haug J.T. (2023). An expanded view on the morphological diversity of long-nosed antlion larvae further supports a decline of silky lacewings in the past 100 million years. Insects.

[74] Mengel L., Linhart S., Haug G.T., Weiterschan T., Müller P., Hoffeins C., Hoffeins H.-W., Baranov V., Haug C., Haug J.T. (2023). The morphological diversity of dragon lacewing larvae (Nevrorthidae, Neuroptera) changed more over geological time scales than anticipated. Insects.

[75] Buchner L., Linhart S., Kalmar G., Arce S., Haug G.T., Haug J.T., Haug C. (2024). New fossil lacewing larvae with trumpet-shaped elongate empodia provide insight into the evolution of this attachment structure. Riv. Ital. Paleontol. Stratigr..

[76] Oswald J.D., Machado R.J.P., Foottit R.G., Adler P.H. (2008). Biodiversity of the Neuropterida (Insecta: Neuroptera, Megaloptera, and Raphidioptera). Insect Biodiversity: Science and Society.

[77] Satar A., Tusun S., Bozdogan H. (2014). Third instars larvae of *Gepus gibbosus* Hölzel, 1968 (Neuroptera: Myrmeleontindae). Zootaxa.

[78] Humeau A., Rougé J., Casas J. (2015). Optimal range of prey size for antlions. Ecol. Entomol..

[79] MacLeod E.G. (1964). A Comparative Morphological Study of the Head Capsule and Cervix of Larval Neuroptera (Insecta). Ph.D. Thesis.

[80] Zimmermann D., Randolf S., Aspöck U., Krenn H.W. (2019). From chewing to sucking via phylogeny—From sucking to chewing via ontogeny: Mouthparts of Neuroptera. Insect Mouthparts: Form, Function, Development and Performance.

[81] New T.R. (1982). The larva of *Nymphes* Leach (Neuroptera: Nymphidae). Neuroptera Internat..

[82] Froggatt W.W. (1907). Australian Insects.

[83] Riek E.F. (1970). Neuroptera (Lacewings). Insects of Australia: A Textbook for Students and Research Workers.

[84] Van der Weele H.W. (1908). Ascalaphiden. Collections Zoologiques du Baron Edm. de Selys Longchamps. Catal. Syst. Descr..

[85] Tillyard R.J. (1926). The Insects of Australia and New Zealand.

[86] Ross E.S. (1953). Insects Close Up: A Pictorial Guide for the Photographer and Collector Featuring 125 Photographs and Drawings.

[87] New T.R. (1983). Some early stages of *Osmylops* (Neuroptera: Nymphidae). Syst. Entomol..

[88] Gepp J. (1984). Erforschungsstand der Neuropteren. Larven der Erde (mit einem Schlüssel zur Larvaldiagnose der Familien, einer Übersicht von 340 beschriebenen Larven und 600 Literaturzitaten). Prog. World’s Neuropterol..

[89] New T.R., Lambkin K.J. (1989). The larva of *Norfolius* (Neuroptera: Nymphidae). Syst. Entomol..

[90] Shi C., Winterton S.L., Ren D. (2015). Phylogeny of split-footed lacewings (Neuroptera, Nymphidae), with descriptions of new Cretaceous fossil species from China. Cladistics.

[91] Schindelin J., Arganda-Carreras I., Frise E., Kaynig V., Longair M., Pietzsch T., Preibisch S., Rueden C., Saalfeld S., Schmid B. (2012). Fiji: An open-source platform for biological-image analysis. Nat. Meth..

[92] Badano D., Aspöck U., Aspöck H., Cerretti P. (2017). Phylogeny of Myrmeleontiformia based on larval morphology (Neuropterida: Neuroptera). Syst. Entomol..

[93] Iwata H., Ukai Y. (2002). SHAPE: A computer program package for quantitative evaluation of biological shapes based on elliptic Fourier descriptors. J. Hered..

[94] Bonhomme V., Picq S., Gaucherel C., Claude J. (2014). Momocs: Outline analysis using R. J. Stat. Softw..

[95] Ax P. (1995). Das System der Metazoa Ein Lehrbuch der phylogenetischen Systematik.

[96] Donoghue P.C. (2005). Saving the stem group—A contradiction in terms?. Paleobiology.

[97] Jefferies R.P.S., House M.R. (1979). The origin of chordates: A methodological essay. The Origin of Major Invertebrate Groups. Systematics Association Special Volume 12.

[98] Liu H., Beutel R.G., Makarov K.V., Jarzembowski E.A., Xiao C., Luo C. (2023). The first larval record of Migadopinae (Coleoptera: Adephaga: Carabidae) from mid-Cretaceous Kachin amber, northern Myanmar. Cretac. Res..

[99] Liu H., Makarov K.V., Jarzembowski E.A., Xiao C., Luo C. (2023). *Cretoloricera electra* gen. et sp. nov., the oldest record of Loricerini (Coleoptera: Adephaga: Carabidae: Loricerinae) from mid-Cretaceous Kachin amber. Cretac. Res..

[100] Rosová K., Prokop J., Hammel J.U., Beutel R.G. (2023). The earliest evidence of Omophroninae (Coleoptera: Carabidae) from mid-Cretaceous Kachin amber and the description of a larva of a new genus. Arthropod Syst. Phyl..

