# Supplementary material for: New Data Indicate Larger Decline in Morphological Diversity in Split-Footed Lacewing Larvae than Previously Estimated
Source: Insects. 2025 Jan 27;16(2):125. doi: 10.3390/insects16020125 (PMC11855922; doi:10.3390/insects16020125)
Supplement: Supplementary file 1 [file insects-16-00125-s001.zip › insects-3012998-supplementary/Suppl Files Analysis Nymphidae/03/Nym_03_Variation.rtf]

    axis proportion cumsum   <int>      <dbl>  <dbl> 1     1    0.438    0.438 2     2    0.183    0.620 3     3    0.128    0.748 4     4    0.0767   0.825 5     5    0.0501   0.875 6     6    0.0279   0.903 7     7    0.0241   0.927 8     8    0.0226   0.950 9     9    0.00945  0.95910    10    0.00894  0.968	
