# Supplementary material for: New Data Indicate Larger Decline in Morphological Diversity in Split-Footed Lacewing Larvae than Previously Estimated
Source: Insects. 2025 Jan 27;16(2):125. doi: 10.3390/insects16020125 (PMC11855922; doi:10.3390/insects16020125)
Supplement: Supplementary file 1 [file insects-16-00125-s001.zip › insects-3012998-supplementary/Suppl Files Analysis Nymphidae/08/Nym_08_Variation.rtf]

	    axis proportion cumsum   <int>      <dbl>  <dbl> 1     1    0.749    0.749 2     2    0.128    0.878 3     3    0.0507   0.929 4     4    0.0312   0.960 5     5    0.0117   	0.971 6     6    0.00804  0.980 7     7    0.00568  0.985 8     8    0.00432  0.990 9     9    0.00359  0.99310    10    0.00223  0.995
