# Supplementary figures and images for: New Data Indicate Larger Decline in Morphological Diversity in Split-Footed Lacewing Larvae than Previously Estimated
Source: Insects. 2025 Jan 27;16(2):125. doi: 10.3390/insects16020125 (PMC11855922; doi:10.3390/insects16020125)

-2S.D.

Mean

+2S.D.

PC1

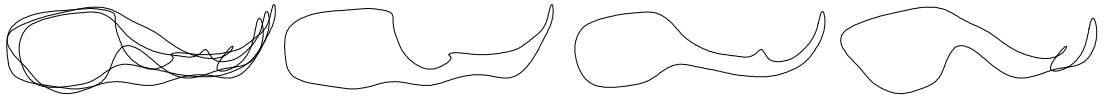

PC2

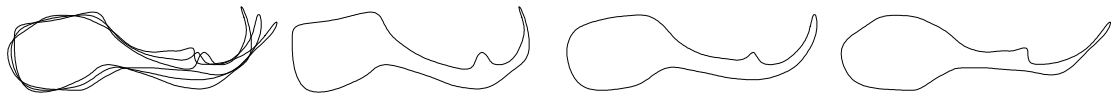

PC3

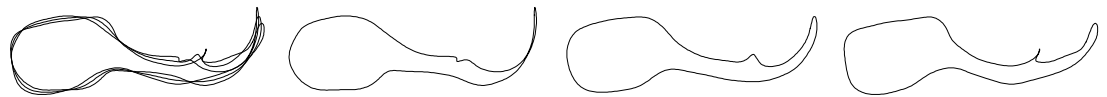

PC4

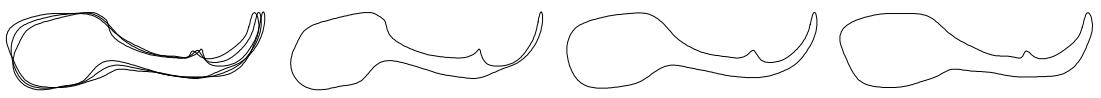

PC5

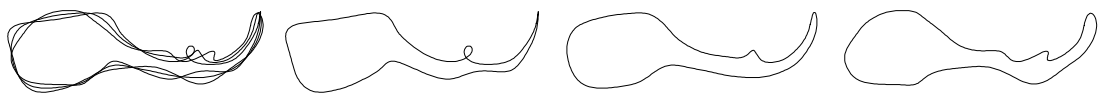

-2S.D.

Mean

+2S.D.

PC6

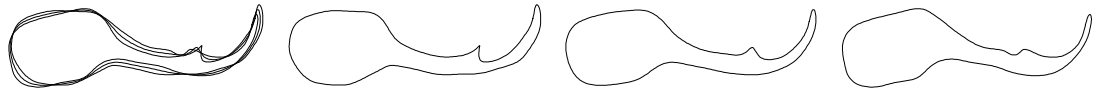

Supplement: Supplementary file 1 [file insects-16-00125-s001.zip › insects-3012998-supplementary/Suppl Files Analysis Nymphidae/01/01.pdf]

-2S.D.

Mean

+2S.D.

PC1

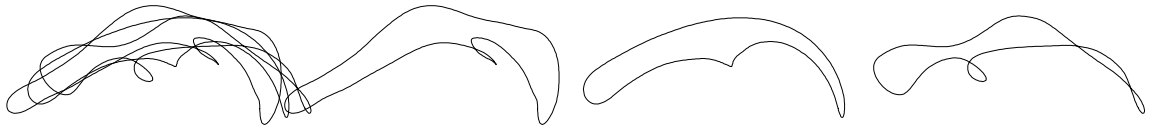

PC2

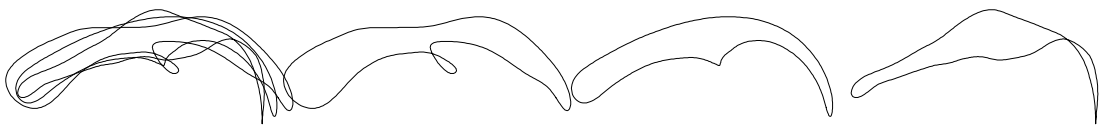

PC3

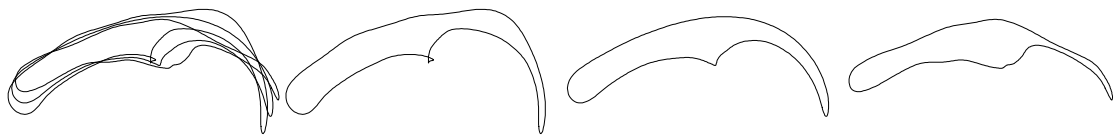

PC4

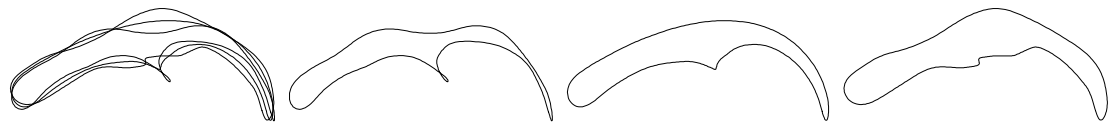

PC5

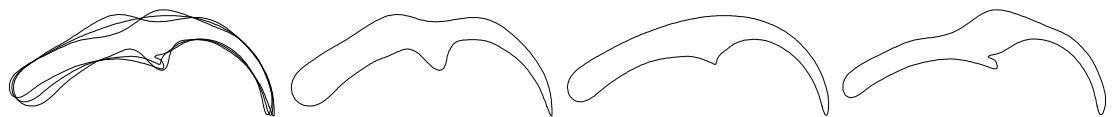

-2S.D.

Mean

+2S.D.

PC6

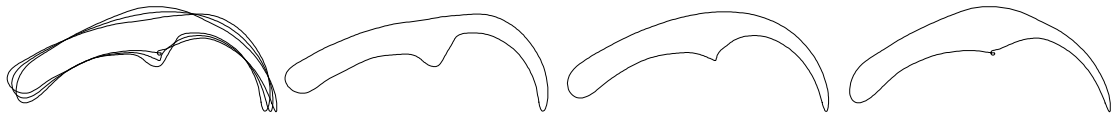

PC7

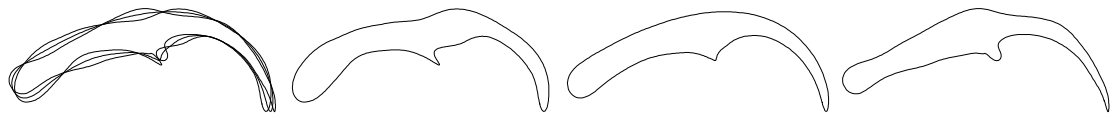

Supplement: Supplementary file 1 [file insects-16-00125-s001.zip › insects-3012998-supplementary/Suppl Files Analysis Nymphidae/02/02.pdf]

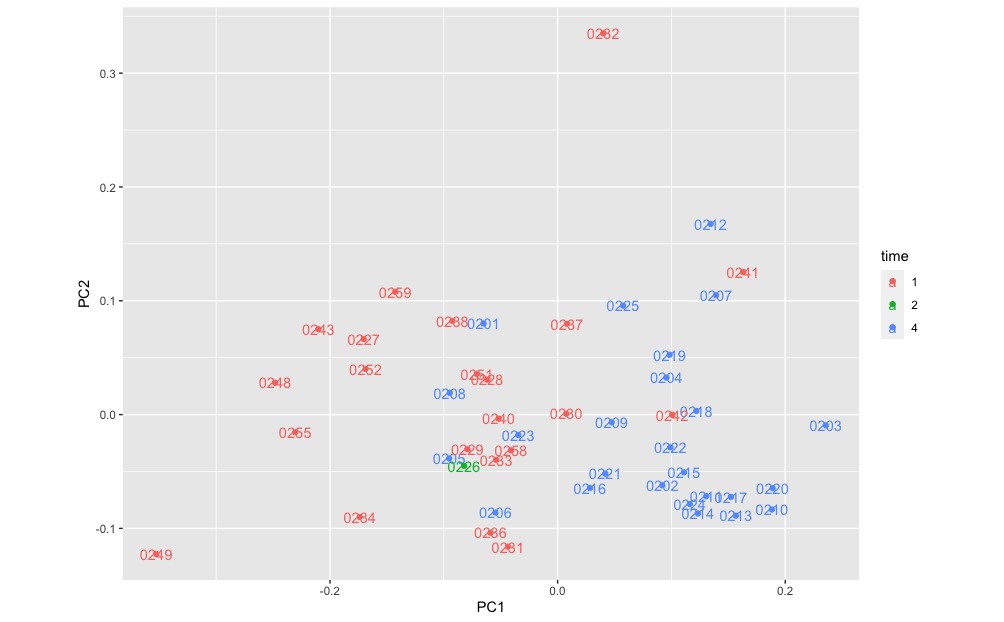

Supplement: Supplementary file 1 [file insects-16-00125-s001.zip › insects-3012998-supplementary/Suppl Files Analysis Nymphidae/03/Nym_03_PCAnummern.jpeg]

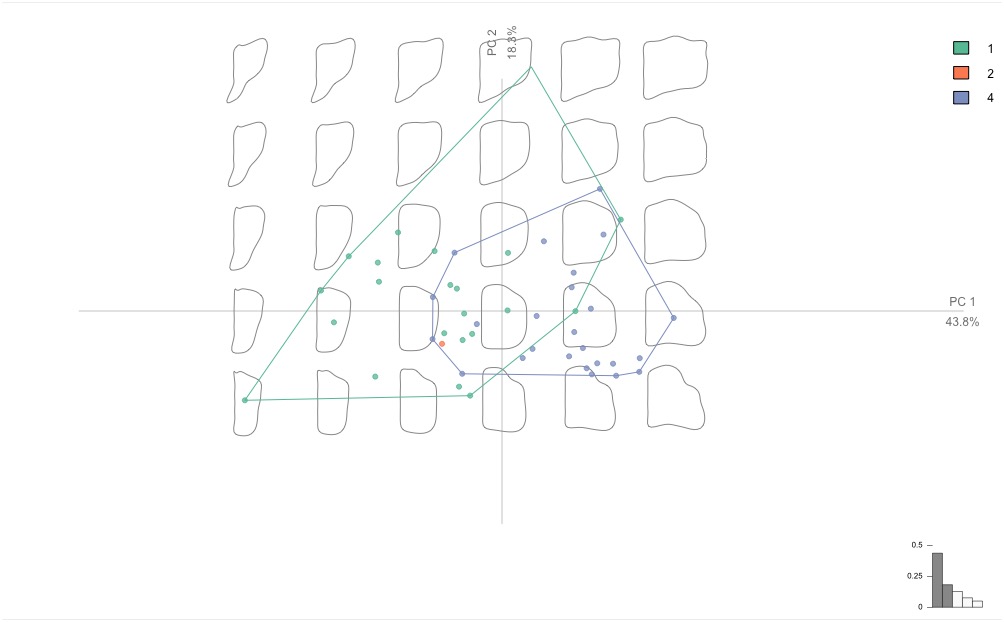

Supplement: Supplementary file 1 [file insects-16-00125-s001.zip › insects-3012998-supplementary/Suppl Files Analysis Nymphidae/03/Nym_03_PCAtime.jpg]

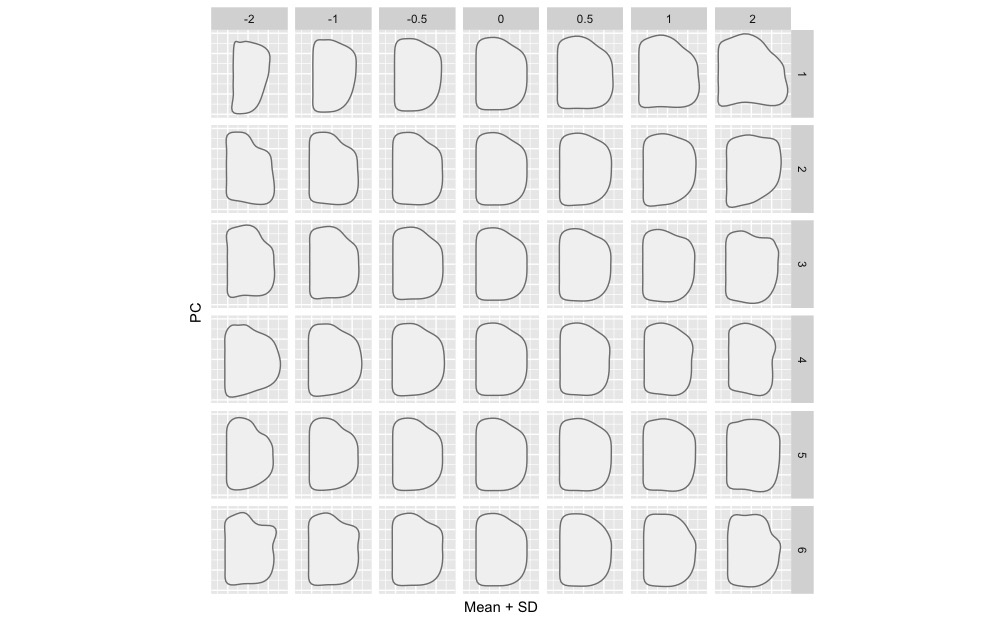

Supplement: Supplementary file 1 [file insects-16-00125-s001.zip › insects-3012998-supplementary/Suppl Files Analysis Nymphidae/03/Nym_03_PCcontrib.jpg]

-2S.D.

Mean

+2S.D.

PC1

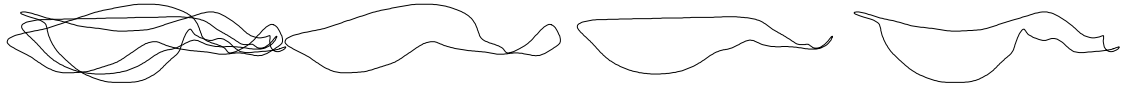

PC2

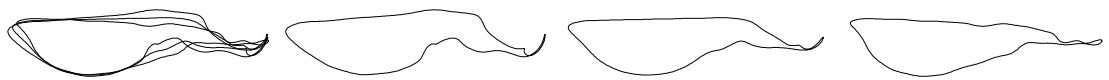

PC3

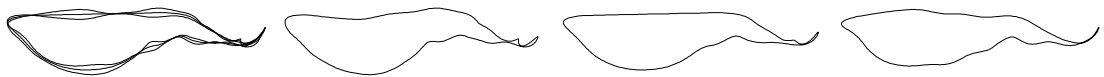

PC4

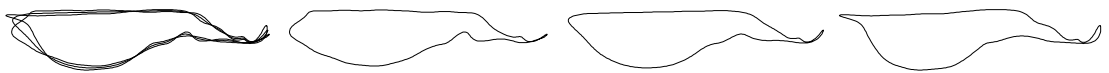

PC5

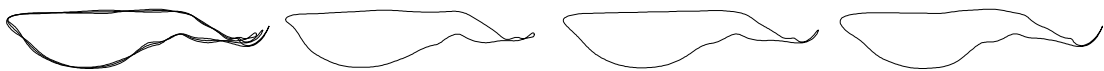

-2S.D.

Mean

+2S.D.

PC6

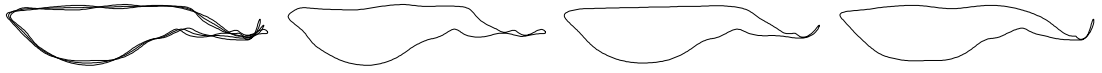

PC7

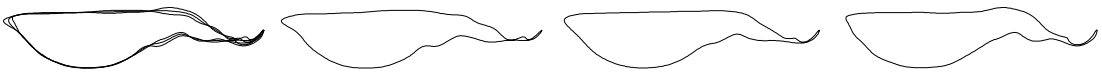

Supplement: Supplementary file 1 [file insects-16-00125-s001.zip › insects-3012998-supplementary/Suppl Files Analysis Nymphidae/04/04.pdf]

-2S.D.

Mean

+2S.D.

PC1

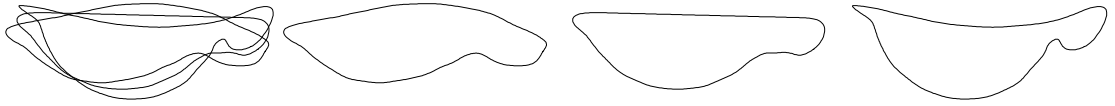

PC2

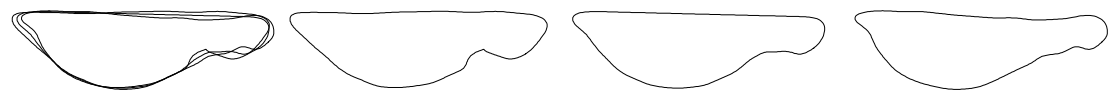

PC3

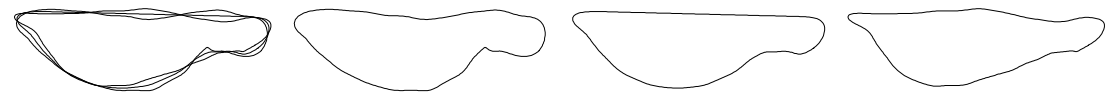

PC4

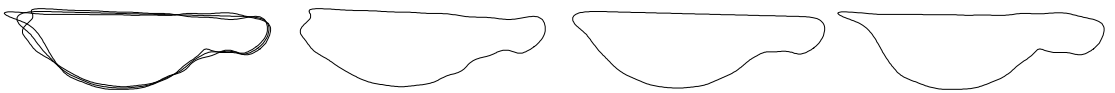

PC5

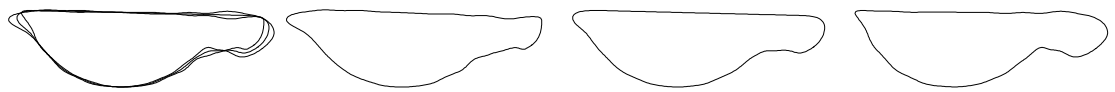

-2S.D.

Mean

+2S.D.

PC6

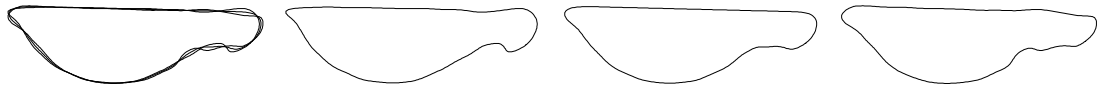

PC7

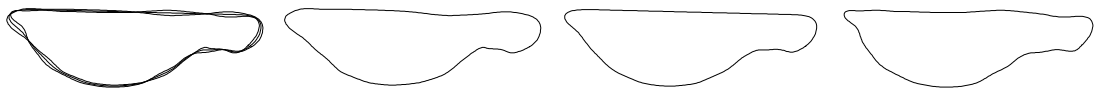

Supplement: Supplementary file 1 [file insects-16-00125-s001.zip › insects-3012998-supplementary/Suppl Files Analysis Nymphidae/05/05.pdf]

-2S.D.

Mean

+2S.D.

PC1

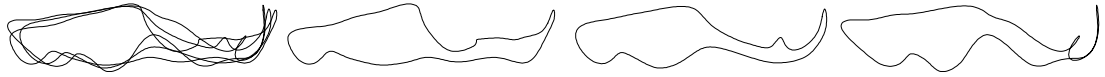

PC2

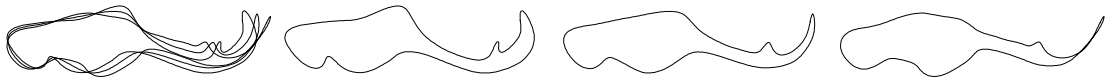

PC3

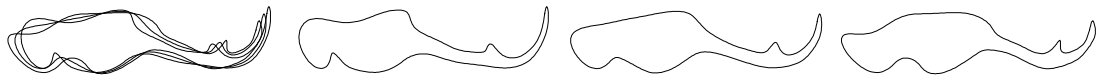

PC4

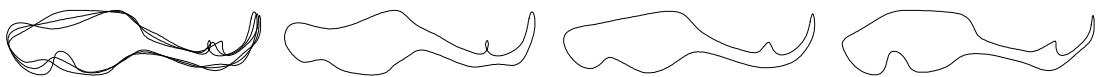

PC5

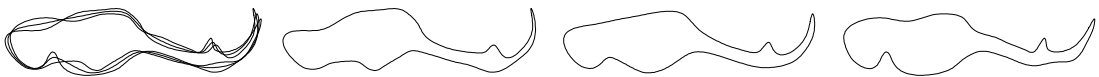

-2S.D.

Mean

+2S.D.

PC6

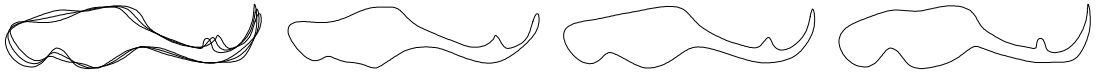

PC7

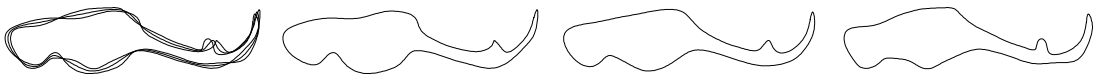

PC8

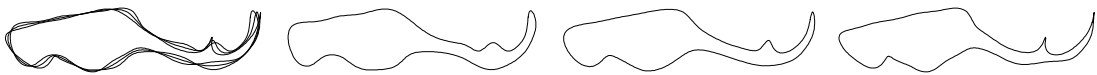

Supplement: Supplementary file 1 [file insects-16-00125-s001.zip › insects-3012998-supplementary/Suppl Files Analysis Nymphidae/06/06.pdf]

-2S.D.

Mean

+2S.D.

PC1

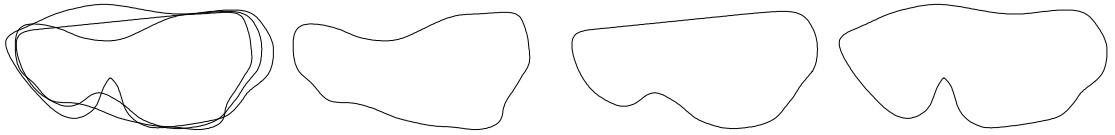

PC2

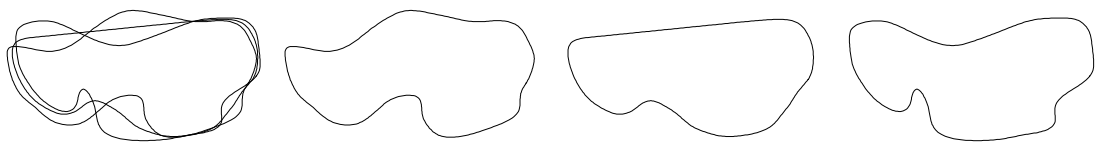

PC3

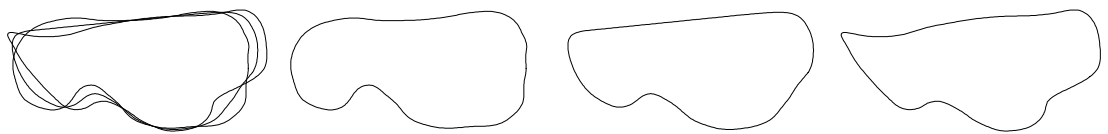

PC4

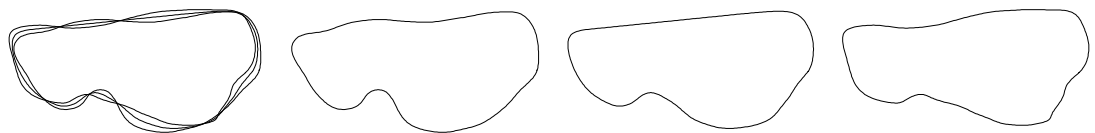

PC5

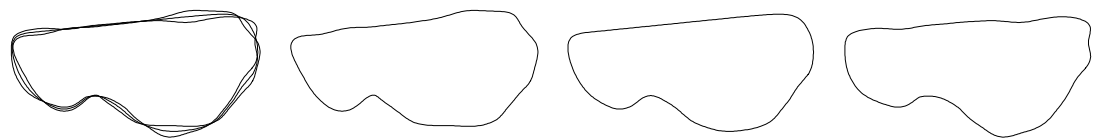

-2S.D.

Mean

+2S.D.

PC6

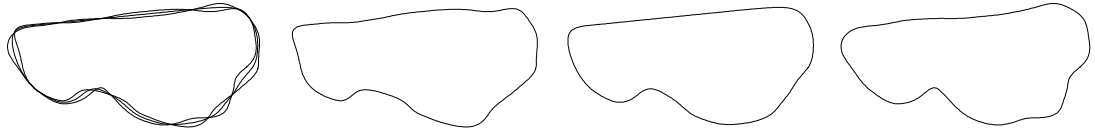

Supplement: Supplementary file 1 [file insects-16-00125-s001.zip › insects-3012998-supplementary/Suppl Files Analysis Nymphidae/07/07.pdf]

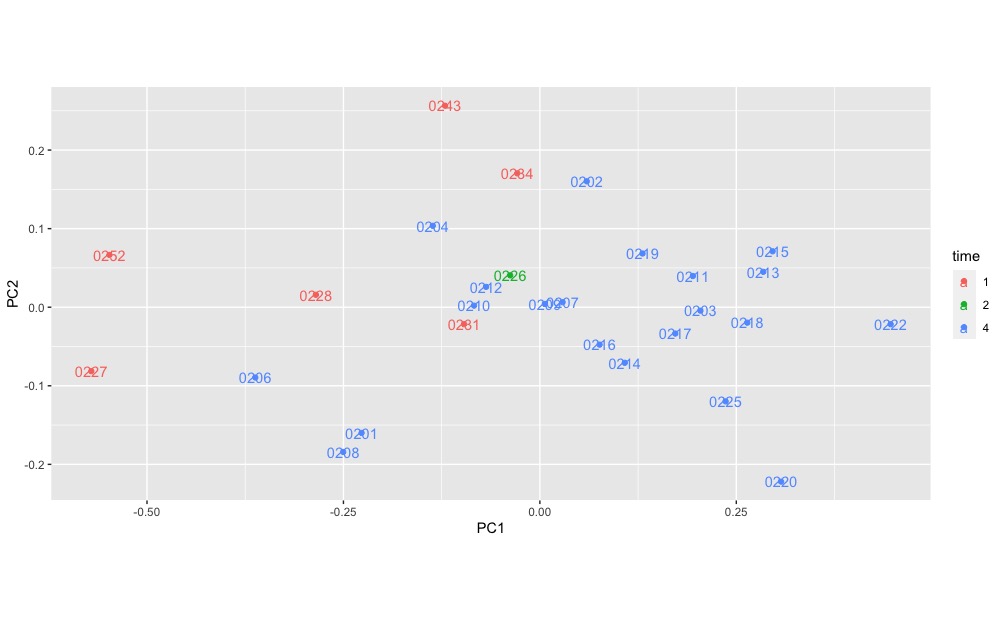

Supplement: Supplementary file 1 [file insects-16-00125-s001.zip › insects-3012998-supplementary/Suppl Files Analysis Nymphidae/08/Nym_08_PCAnummern.jpeg]

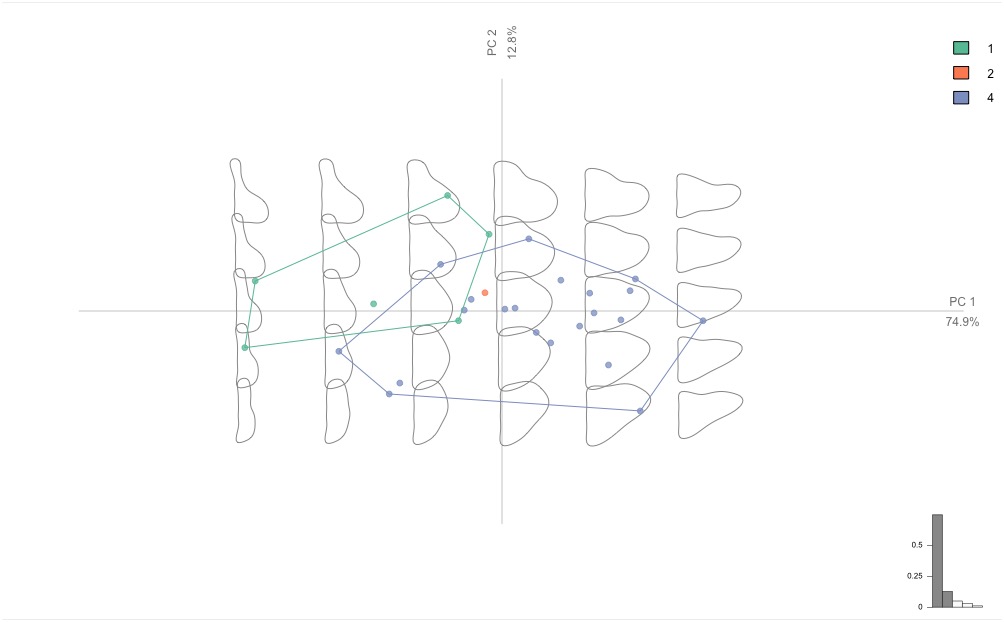

Supplement: Supplementary file 1 [file insects-16-00125-s001.zip › insects-3012998-supplementary/Suppl Files Analysis Nymphidae/08/Nym_08_PCAtime.jpg]

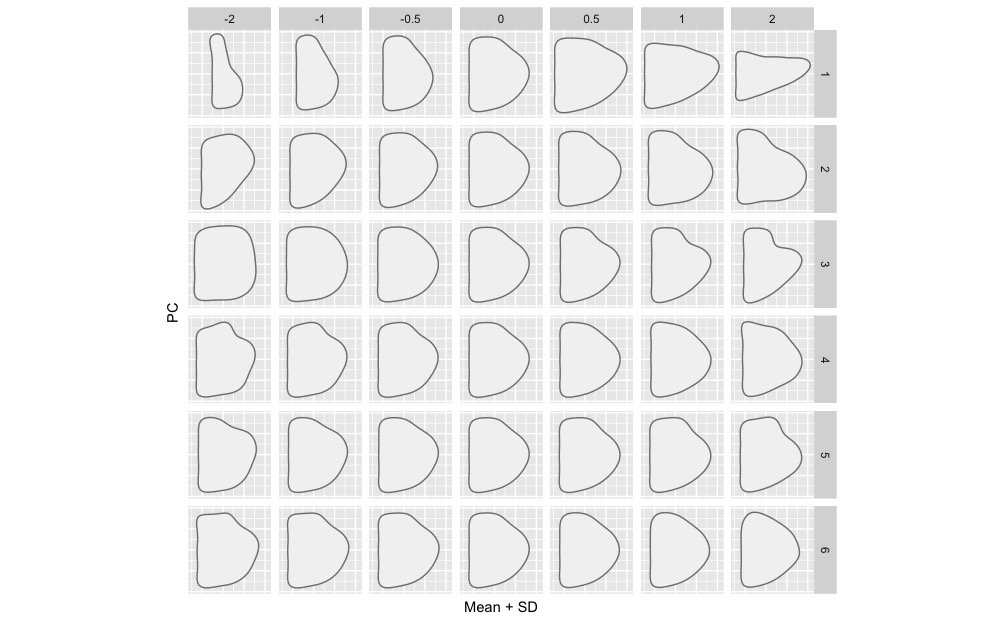

Supplement: Supplementary file 1 [file insects-16-00125-s001.zip › insects-3012998-supplementary/Suppl Files Analysis Nymphidae/08/Nym_08_PCcontrib.jpg]

-2S.D.

Mean

+2S.D.

PC1

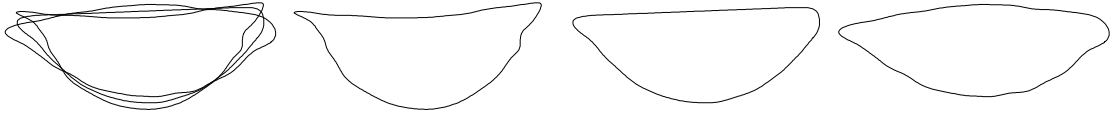

PC2

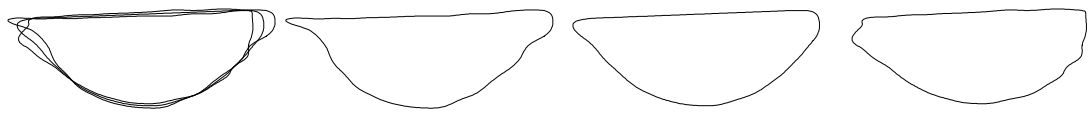

PC3

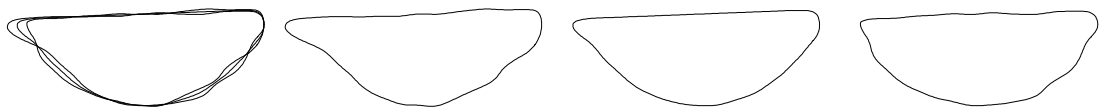

PC4

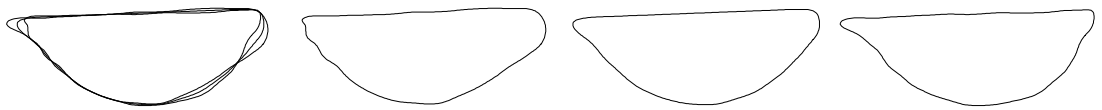

PC5

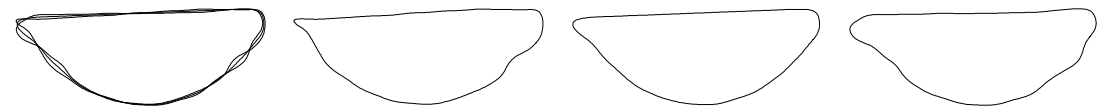

-2S.D.

Mean

+2S.D.

PC6

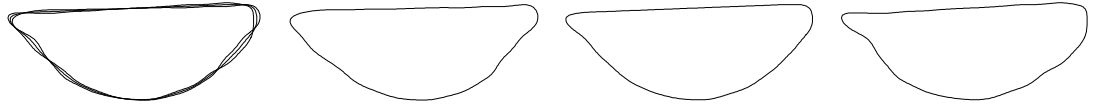

PC7

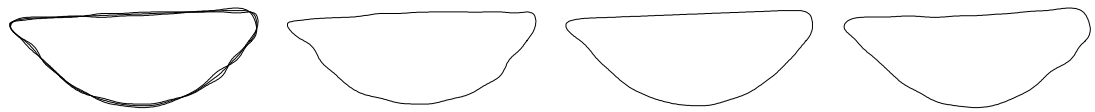

Supplement: Supplementary file 1 [file insects-16-00125-s001.zip › insects-3012998-supplementary/Suppl Files Analysis Nymphidae/09/09.pdf]

-2S.D.

Mean

+2S.D.

PC1

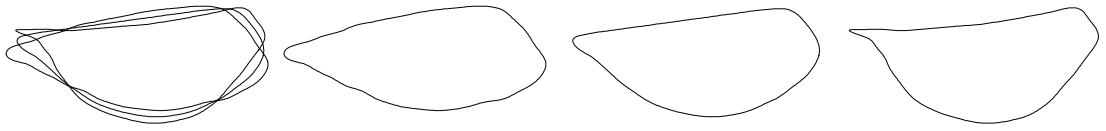

PC2

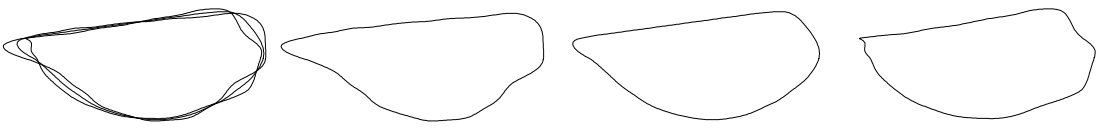

PC3

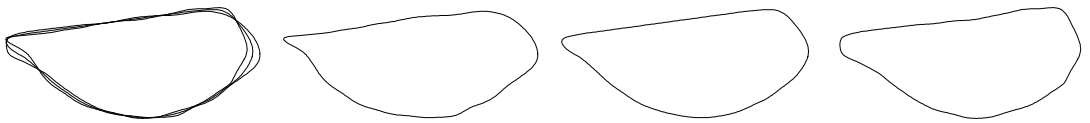

PC4

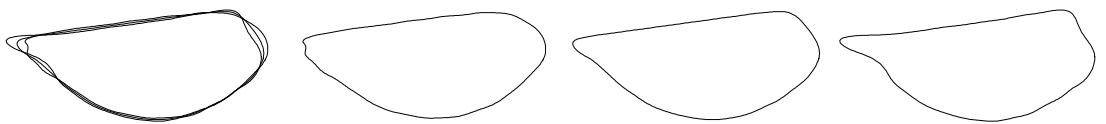

PC5

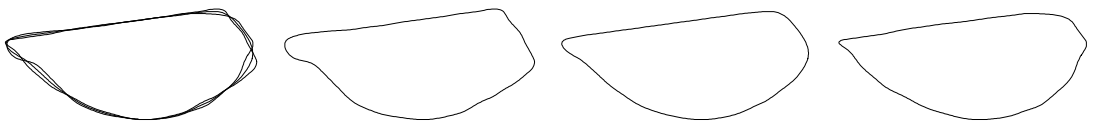

-2S.D.

Mean

+2S.D.

PC6

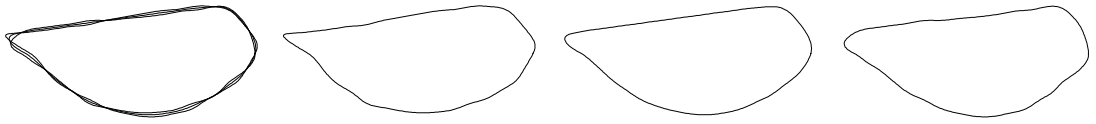

PC7

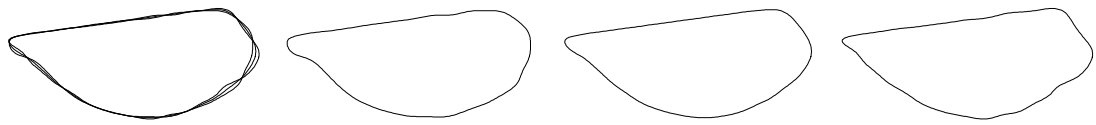

Supplement: Supplementary file 1 [file insects-16-00125-s001.zip › insects-3012998-supplementary/Suppl Files Analysis Nymphidae/10/10.pdf]
